# Long‐Term Memory Updating Parallels Altered Awake and Sleep Hippocampal Replays

**DOI:** 10.1002/advs.202416480

**Published:** 2025-11-05

**Authors:** Jifu Tong, Yuanwei Xing, Yawen Zheng, Linshu Wang, Shan Shao, Jiao Wu, Longyu Ma, Shuting Liu, Naizheng Liu, Xuetao Qi, Ting Wang, Kun Cui, Shuang Cui, You Wan, Ming Yi

**Affiliations:** ^1^ Neuroscience Research Institute and Department of Neurobiology School of Basic Medical Sciences Peking University Beijing 100083 P. R. China; ^2^ Beijing Life Science Academy Beijing 102209 P. R. China; ^3^ Key Laboratory for Neuroscience Ministry of Education / National Health Commission Peking University Beijing 100083 P. R. China; ^4^ Medical Innovation Center (Taizhou) of Peking University Taizhou 225316 P. R. China

**Keywords:** hippocampal place cells, replays, sharp wave‐ripples, memory updating, sleep

## Abstract

The activity of hippocampal place cells is essential for various processes of spatial memory, including encoding, consolidation, and retrieval. However, whether they also participate in spatial memory updating remains elusive. This study demonstrates that during the process of long‐term spatial associative memory updating, triggered by changes in reward magnitudes in a maze over consecutive days, there is a distinct period characterized by loss of preference for different reward magnitudes before adapting to new magnitude ratios. During this period, sequential replays in the hippocampus during post‐training sleep are significantly biased toward the region associated with the larger reward. Additionally, replays between trials show elevated predictive power for the upcoming choices. Our results suggest that hippocampal replays may play a key role in updating long‐term spatial associative memory.

## Graphical abstract

This study introduces a behavioral paradigm for memory updating and reveals underlying hippocampal mechanisms. Replays of place cells predict future behavioral choices during behavior and show a bias toward high‐reward locations during non‐rapid eye movement (NREM) sleep, specifically within the memory updating process. These findings suggest that hippocampal dynamics play a critical role in integrating new information into existing memories.

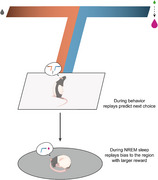

## 1 Introduction

Memory is a fundamental cognitive ability that enables animals to encode, store, and retrieve information over time.^[^ ^1^, ^2^ ^]^ However, memories are not static. When new experiences differ from memory‐based expectations, the contents of memories can be updated according to this prediction error, ensuring that memory remains flexible and aligned with current realities.^[^ ^3^ ^]^

The hippocampus plays a crucial role in memory,^[^ ^4^ ^]^ with place cells serving as the neuronal basis of internal cognitive maps.^[^ ^5^, ^6^ ^]^ During consumptive behavior, awake immobility, and sleep, high‐frequency oscillation events named sharp wave‐ripples (SWRs) emerge in the hippocampal CA1 region (CA1) local field potential (LFP), accompanied by simultaneous bursts of CA1 neuronal populations.^[^ ^7^, ^8^ ^]^ During these population burst events (PBEs), place cells are activated. Within some PBEs, place cells are activated in sequences resembling those observed during prior experience, which is called replay.^[^ ^9^, ^10^, ^11^, ^12^, ^13^ ^]^ These sequential replays of place cells are significantly associated with hippocampal memory functions. Sleep replays following training have been strongly linked to memory consolidation,^[^ ^14^, ^15^, ^16^ ^]^ whereas recent studies suggest that awake replays are involved in memory retrieval^[^ ^17^, ^18^ ^]^ and planning of future behaviors.^[^ ^19^, ^20^ ^]^ However, while hippocampal replays are well‐documented in various memory processes, their role in memory updating remains less well understood.

In the present study, we developed a behavioral task to investigate the updating process of long‐term spatial associative memory. Rats were permitted to make free choices to obtain larger rewards as the reward magnitudes changed over the course of several days. We recorded activities of hippocampal place cells in the dorsal CA1 region during exploration, awake rest, and sleep, and examined whether the activities of place cells, especially replays, during reward consumption, awake rest, and sleep, were associated with the process of memory updating.

Our results indicate that memory updating in animals undergoes a two‐stage process: an initial loss of long‐term preference, followed by behavioral adaptation to new experiences. During this transition, replay events during non‐rapid eye movement (NREM) sleep exhibit a bias toward the newly preferred region, whereas replays during awake rest do not. Additionally, replays during behavior acquire predictive power for the animals' future choices, specifically during the memory updating period. These findings suggest that hippocampal replay across different behavioral states contributes to long‐term memory updating through distinct mechanisms. Collectively, these results advance our understanding of the state‐dependent functional heterogeneity of hippocampal replay in adaptive memory processes.

## 2 Results and Discussion

### 2.1 Rats Update Their Memory Upon Reward Magnitude Change

**Figure 1:**
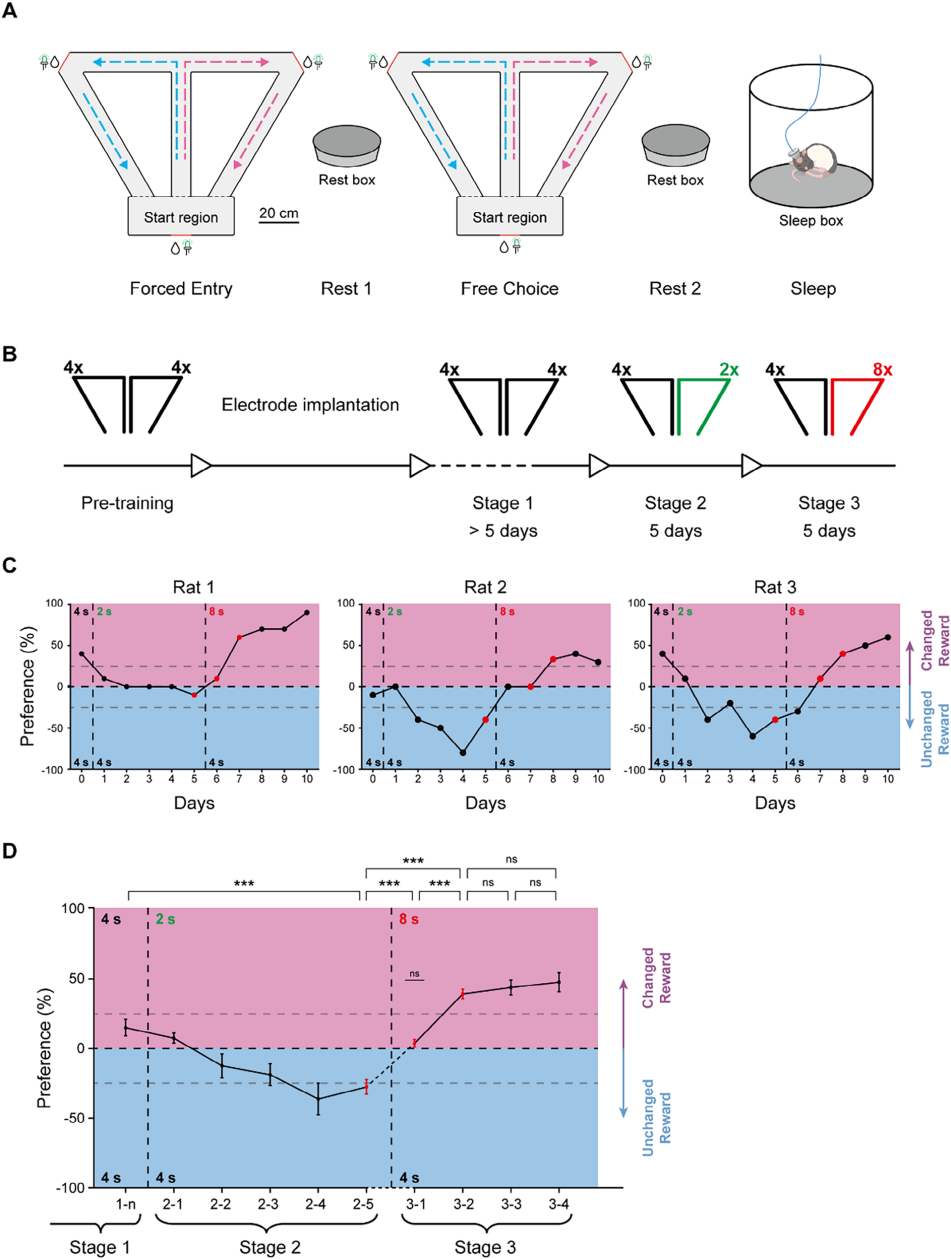
Rats update their memory upon reward changes. A) Behavioral apparatus and recording procedure. From left to right: Forced Entry session in the maze; Rest 1 session in the rest box; Free Choice session in the maze; Rest 2 session in the rest box; Sleep session in the sleep box. Scale bar, 20 cm. B) Experimental procedures. From left to right: (1) pre‐training with 4‐s versus 4‐s reward ratio for ≈7 days; (2) electrode implantation, recovery and tetrode driving lasting 2–3 weeks; (3) Stage 1 with re‐training under 4‐s versus 4‐s reward ratio for more than 5 days; (4) Stage 2 with 4‐s versus 2‐s reward ratio for 5 consecutive days; (5) Stage 3 with 4‐s versus 8‐s reward ratio for 5 days. C) Representative behavioral performances. Purple region: preference to the changed reward side; blue region: preference to the unchanged reward side. Horizontal gray dashed lines indicate the boundary of clear preferences (±25%). Vertical dashed lines indicate the boundary between stage 1, stage 2, and stage 3. Red dots indicate consolidated preference period (CON), losing preference period (LOS), and reversing preference period (REV) periods. D) The preferences of all rats throughout the memory updating paradigm, in which the days in stage 3 are aligned with respect to the REV period as the reference point. One‐way ANOVA with Tukey post hoc analysis: F (9, 70) = 21.00, *p <* 10^−4^, day 1‐n versus day 2‐5: *p =* 8 × 10^−4^; day 2‐5 versus day 3‐1: *p =* 0.0394; day 2‐5 versus day 3‐2: *p <* 10^−4^; day 3‐1 versus day 3‐2: *p =* 0.0106; day 3‐2 versus day 3‐3: *p >* 0.9999; day 3‐2 versus day 3‐4: *p =* 0.9962; day 3‐3 versus day 3‐4: *p >* 0.9999. One sample t‐test: day 3‐1 versus 0: *p =* 0.1970. Data are presented as mean ± SEM (n = 8 rats). ***p < 0.001; **p < 0.01; *p < 0.05; ns, *p >* 0.05.

To explore the mechanisms underlying the updating of long‐term spatial associative memory, we designed a T‐maze‐based behavioral task in which rats were trained to seek larger sucrose water rewards (**Figure** **1**A). The maze contained three water ports in the Start Region and at both terminals of the horizontal arm, which deliver sucrose water at the same velocity. We utilized sucrose water delivery of 2, 4, and 8 s as the relatively small, medium, and large rewards, respectively.

Following a period of pre‐training, rats participated in a structured daily behavioral protocol comprising five consecutive sessions: Forced Entry, Rest 1, Free Choice, Rest 2, and Sleep. During the Forced Entry and Free Choice sessions, rats initiated each trial from the start region, traversed the central arm of a T‐maze, and selected either the left or right horizontal arm to receive a water reward. They subsequently returned along the returning arm of the same side to obtain an additional reward before commencing the next trial. In the Forced Entry sessions, the animals’ choices were directed by a guiding light located at one of the two horizontal arms, whereas in the Free Choice sessions, rats were allowed to freely select each side. The magnitude of the water reward was held constant across both session types within each day. The Forced Entry session continued for either 30 trials or 30 min, whichever occurred first, while the Free Choice session terminated after 20 trials or 30 min under the same condition. Each running session was immediately followed by a 20‐min rest session (Rest 1 following Forced Entry and Rest 2 following Free Choice), during which animals were placed in an isolated rest box (Figure S3A, Supporting Information). Upon completion of the two behavioral and two rest sessions, rats were returned to a sleep box, which also served as their home cage throughout the experiment (Figure 1A; Figure S3B, Supporting Information).

The procedure of memory updating comprised three stages: in stage 1, rats were trained to perform both the Forced Entry and Free Choice sessions with a reward ratio of 4‐s versus 4‐s; in Stage 2, rats learned a 4‐s versus 2‐s reward ratio for five consecutive days; and in Stage 3, rats were exposed to a 4‐s versus 8‐s reward ratio to perform memory updating (Figure 1B). The rats’ preferences during the Free Choice sessions were considered as indicators of long‐term memory about rewards, and the transition in the rats' preferences between stage 2 and stage 3 reflected the updating of long‐term spatial associative memory.

By day 5 of Stage 2, rats exhibited a long‐term memory of this reward magnitude ratio, as indicated by their preferences for the side associated with the 4‐s reward on day 5 (consolidated preference period, CON) (4 rats exhibited clear preference to the 4‐s reward larger than 25%, 4 rats exhibited mild preference between 0 and 25%) (Figure 1C,D; Figure S1A, Supporting Information). It was observed that during the transition from Stage 2 to Stage 3, the rats did not immediately reverse their preferences on the first day of Stage 3. Each rat experienced a period with no significant preference for either side, defined as the preferences range between −25% and 25% (Figure S1B, Supporting Information), which occurred on the first or the second day of Stage 3 (for example, day 6 for Rat 1 & 3, day 6‐7 for Rat 2) (Figure 1C; Figure S2, Supporting Information), following which there was a period with clear preferences for the new and larger reward side (preferences larger than 25%, day 7‐10 for Rat 1 and day 8‐10 for Rat 2 & 3) (Figure 1C). To more clearly compare the periods with and without preferences in Stage 3 and to eliminate the differences in memory updating speed among different animals, we aligned the first day when a clear preference for the new and larger reward side emerged in Stage 3. After alignment, we found that following the reward amount change in Stage 3, the rats initially entered a brief phase where they showed no clear preference for either side. Subsequently, the rats developed a clear preference for the new and larger reward side, and this preference remained relatively stable over the following 3 days. Therefore, we defined the first day on which the rats exhibited a clear preference for the new and larger reward side as the reversing preference period (REV period) and the day before the REV period as the losing preference period (LOS period). Preferences for the two reward sides were significantly changed across CON, LOS, and REV periods (Figure 1D). These results showed that memory updating indicated by preference changes occurred primarily within these three periods, especially in the LOS period. Consequently, our subsequent analyses focused on these three critical periods.

### 2.2 Constructing Place Cell Templates for Different Maze Regions

**Figure 2:**
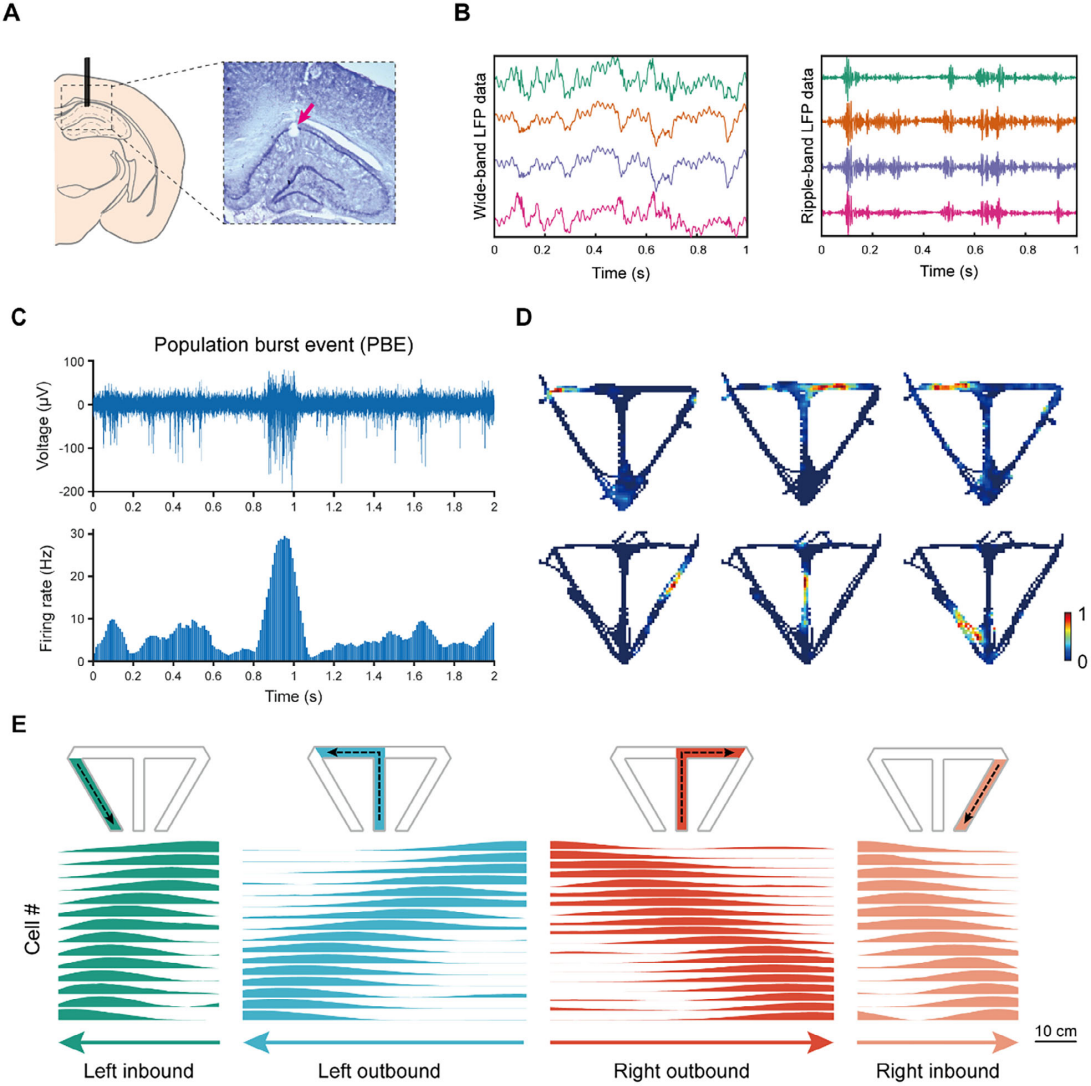
Firing rate templates of each trajectory decode PBEs. A) Position of tetrode tips in the CA1 region. Arrow, electrolytic lesion in the dorsal CA1. B) Representative ripples in the CA1 region. When the tetrodes reached the CA1 region, recognizable SWRs appeared. Left, wide‐band LFP data of 4 channels from different tetrodes; right, the same data filtered with ripple band (100 – 250 Hz). C) Representative PBEs. Upper: 300 Hz high‐pass filtered data of a single channel; lower: firing rate of MUA during the same period. D) Representative 2D firing rate maps of place cells. E) Templates of 1D place cell firing rate maps corresponding to four segments of the running track: left inbound, left outbound, right outbound, and right inbound. The firing rate maps were sorted based on the location of the peak firing rates. Arrows indicate the directions of the segments. Scale bar, 10 cm.

To investigate the activities in the hippocampus throughout the memory updating procedure, we performed in vivo electrophysiological recording in the dorsal CA1 region by implanting custom microdrives consisting of 16 movable tetrodes into the unilateral or bilateral hippocampus (**Figure** **2**A; Figure S4A, Supporting Information). During reward consumption, awake immobility, and NREM sleep, the hippocampus exhibited characteristic SWR events in LFP (Figure 2B). These SWRs were accompanied by PBEs in the CA1 region, characterized by synchronized bursts of CA1 neurons. During PBEs, place cells in CA1 were activated, enabling PBEs to encode spatial information.^[^ ^17^, ^19^, ^21^ ^]^ To identify these critical electrophysiological events, we detected PBEs by thresholding the overall firing rate of the multi‐unit activity (MUA) of all CA1 units (Figure 2C). In parallel with electrophysiological recordings, we conducted video recordings of the animals' behavior on a linear track. The animals' head trajectories and head orientations were extracted using a custom algorithm based on the You Only Look Once (YOLO) model. (Figure S1C–E, Supporting Information).

Previous studies have demonstrated a strong association between online theta oscillations and cognitive processes related to learning and memory.^[^ ^22^, ^23^, ^24^ ^]^ Therefore, we first examined whether relative theta power during running in the maze changed throughout memory updating. The results showed that the relative theta‐band power during running across the three critical periods was not significantly changed throughout the CON, LOS, and REV periods (Figure S4B–D, Supporting Information). These results suggested that theta oscillations per se remain constant throughout memory updating.

A total of 1816 place cells were recorded from six rats, and their spatial firing patterns were represented as 2D firing rate maps (Figure 2D; Table S1, Supporting Information). Given that the maze used in the experiment was functionally 1D, all subsequent analyses were conducted using 1D firing rate maps. To assess the stability of place cell firing across sessions, Pearson correlation coefficients were calculated between the firing rate maps obtained during the Forced Entry and Free Choice sessions. The analysis revealed that the majority of place cells exhibited stable firing patterns across these two sessions, and this stability was consistent across the CON, LOS, and REV experimental periods (Figure S4E,F, Supporting Information). For subsequent analyses, we utilized composite firing rate maps that integrated data from both Forced Entry and Free Choice sessions, excluding cells that exhibited remapping between the two running sessions. Following a methodology adapted from previous studies,^[^ ^25^ ^]^ we segmented the rats' running trajectories into four distinct segments—left inbound, left outbound, right outbound, and right inbound—based on their movement paths and reward consumption. Place cells were then assigned to corresponding trajectory segments according to the distribution of their 1D firing rate map, enabling the construction of place cell templates for each segment (Figure 2E; Figure S4G,H, Supporting Information). Subsequent analyses of place cell activities were performed using these templates.

### 2.3 Activation Preference During Sleep Change in the LOS Period

Previous studies have demonstrated the critical role of hippocampal activity during offline states following behavior—including awake rest and sleep—in memory consolidation.^[^ ^26^, ^27^, ^28^ ^]^ Given the reported similarities between the mechanisms of memory consolidation and memory updating,^[^ ^29^, ^30^ ^]^ we focused on the neural activity of the CA1 region during offline states, including Rest 1, Rest 2, and Sleep sessions.

**Figure 3:**
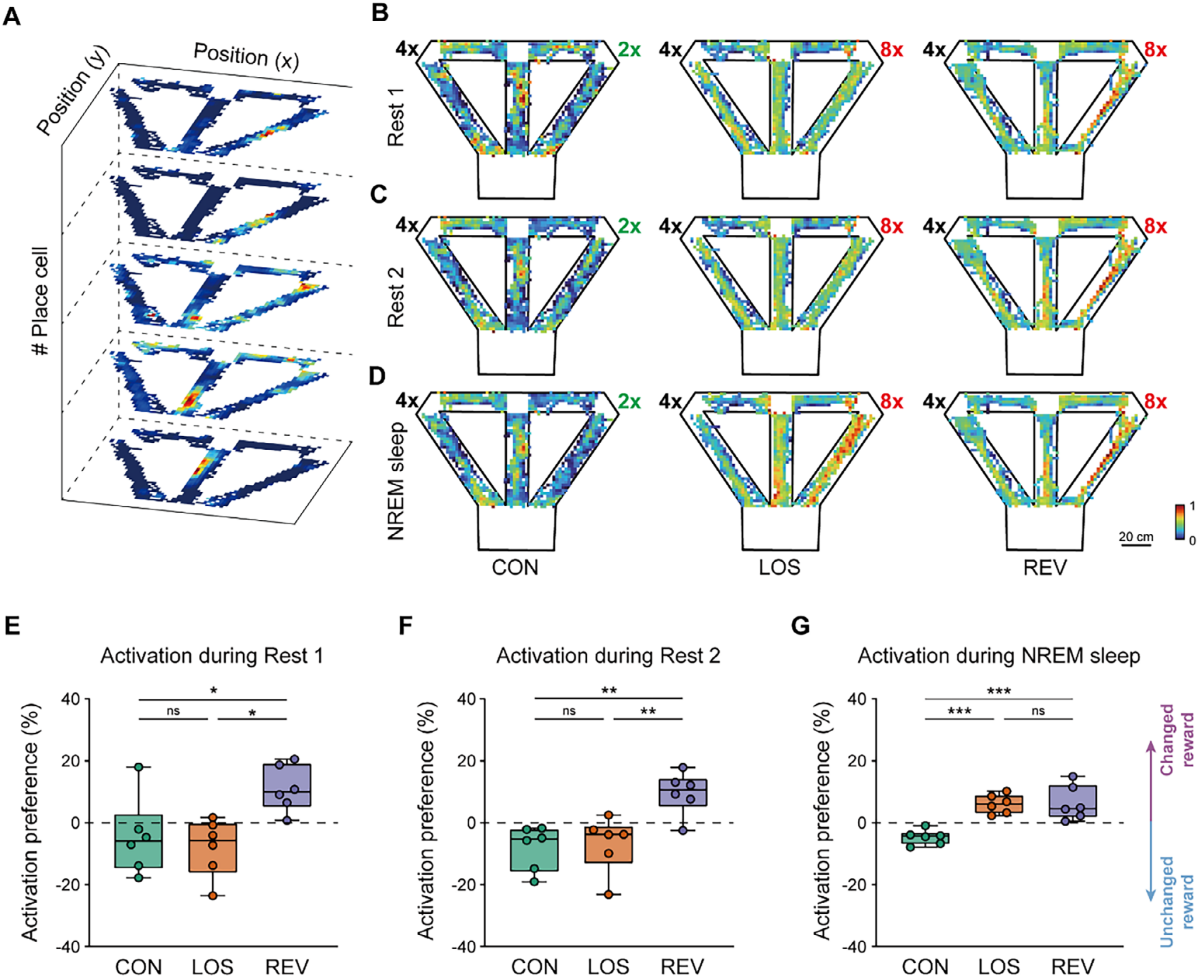
Activation of place cells during sleep is biased toward larger rewards. A) The process of generating a spatial representation map by superimposing 2D firing rate maps of place cells. The weight of each firing rate map corresponds to the activation times of the place cell during PBEs within a specific time period, and the superimposed spatial representation map was then normalized to the range between 0 and 1. B–D) Representative 2D spatial representation maps within PBEs during Rest 1 session (B), Rest 2 session (C), and NREM sleep (D) of CON, LOS, and REV periods (from left to right). Scale bar, 20 cm. E–G) Regional preferences of place cell activations during PBEs in Rest 1 session (E), Rest 2 session (F), and NREM sleep (G) of CON, LOS, and REV period. One‐way ANOVA with Tukey post hoc analysis, Rest 1 session: F (2, 15) = 6.185, *p =* 0.0110, CON versus LOS: *p =* 0.8313, CON versus REV: *p =* 0.0402, LOS versus REV: *p =* 0.0128; Rest 2 session: F (2, 15) = 9.669, *p =* 0.0020, CON versus LOS: *p =* 0.9522, CON versus REV: *p =* 0.0035, LOS versus REV: *p =* 0.0063; NREM sleep: F (2, 15) = 15.27, *p =* 2 × 10^−4^, CON versus LOS: *p =* 8 × 10^−4^, CON versus REV: *p =* 6 × 10^−4^, LOS versus REV: *p =* 0.9895. The center line indicates the median, the box represents the 25th–75th percentiles, and the whiskers show the minimum and maximum values (n = 6 rats). ***p < 0.001; **p < 0.01; *p < 0.05; ns, *p >* 0.05.

We first investigated neural activities during the two rest sessions by computing ripple rates for each rest session across days. The results showed no significant change in ripple rates in either the Rest 1 session or Rest 2 session (Figure S6A,B, Supporting Information). Additionally, we found that PBE rates during Rest 1 and Rest 2 sessions remained relatively stable throughout memory updating (Figure S6C,D, Supporting Information). To investigate the distribution of spatial representation of place cells during offline states, we generated spatial representation maps by superimposing 2D firing rate maps of place cells with the weight of activation frequency within PBEs during Rest 1, Rest 2, and Sleep sessions. The spatial representation map illustrated the regions that were represented during the PBEs in different offline states (**Figure** **3**A–D). The maps suggested that, during PBEs in awake rest sessions, place cells representing the changed reward side tended to be more active than those representing the unchanged reward side in the REV periods (Figure 3B,C). To quantify the distribution of the places represented by the activated place cells, we calculated activation rates of place cells corresponding to left turns (left outbound and left inbound) and right turns (right outbound and right inbound), and computed the preference for the changed reward side in these activations (Figure 3E,F). The results showed that the activation preferences to the changed reward side during both Rest 1 and Rest 2 sessions were not significantly changed in the LOS period compared to the CON period, while the activation preferences were significantly increased in the REV period. These results indicated that the place cell activation during awake rests in the memory updating period does not reflect the changed relation of reward amounts immediately in Stage 3.

Next, we paid attention to the place cell activations during sleep. To explore this, we recorded electrophysiological data from the CA1 region during Sleep sessions following the completion of the four‐session recordings. Previous studies have shown that CA1 cell activation predominantly occurs during NREM sleep stages.^[^ ^21^, ^31^ ^]^ Thus, we extracted the NREM stages based on the delta and theta band power of the LFP data and the movement of the rat's head (Figure S5A,B, Supporting Information). We first investigated whether sleep architecture was influenced by the memory updating process. The results showed that the proportion of sleep within the initial 2 h of the Sleep session remained statistically unchanged throughout the memory updating process (Figure S5E, Supporting Information). And the proportion of NREM sleep within the first 30 min of sleep showed no significant change during the memory updating process (Figure S5F, Supporting Information). Additionally, the PBE rate during NREM sleep exhibited no significant change throughout the memory updating process (Figure S5G, Supporting Information). Then we analyzed the activation preferences during NREM sleep. The results showed that activation preferences during NREM sleep were significantly increased in the LOS period (Figure 3G), consistent with the trend observed in the spatial representation map (Figure 3D), which may support the reconsolidation of new memory content during memory updating. These results indicated that activation preferences of place cells during awake rests do not reflect the changed experiences in the LOS period, whereas activation preferences during sleep do. Activation of hippocampal place cells reflected the overall neuronal representation of spatial locations. Therefore, our findings suggested that although previous studies have emphasized the important role of awake rest in memory consolidation during initial learning,^[^ ^32^ ^]^ awake rest may be less critical than sleep for the updating of long‐term memories.

### 2.4 Replays During Sleep Bias Toward Larger Rewards

**Figure 4:**
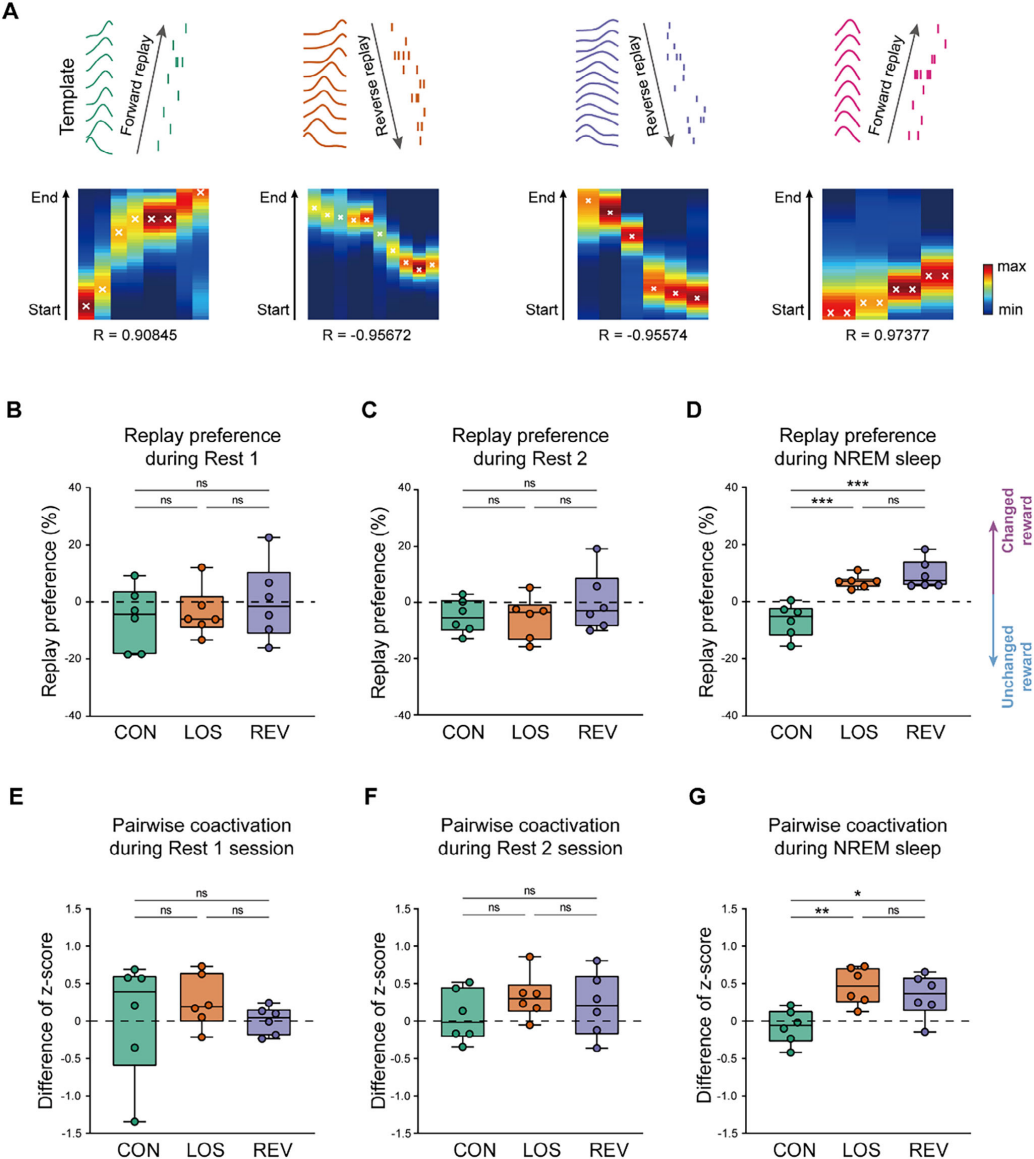
Replays during sleep bias toward larger rewards. A) Four representative replays corresponding to four templates. In each subfigure, the upper left shows the rate maps of place cells activated during the PBEs, the upper right is a raster plot of place cell spikes, and the lower is the distribution of possibilities calculated by Bayesian decoding, along with the corresponding correlation coefficient (R value). B–D) Regional preferences of replays during Rest 1 session (B), Rest 2 session (C), and NREM sleep (D). One‐way ANOVA with Tukey post hoc analysis, Rest 1 session: F (2, 15) = 0.4037, *p =* 0.6749, CON versus LOS: *p =* 0.9518, CON versus REV: *p =* 0.6585, LOS versus REV: *p =* 0.8308; Rest 2 session: F (2, 15) = 0.8003, *p =* 0.4675, CON versus LOS: *p =* 0.9949, CON versus REV: *p =* 0.5616, LOS versus REV: *p =* 0.5049; NREM sleep: F (2, 15) = 20.30, *p <* 10^−4^, CON versus LOS: *p =* 4 × 10^−4^, CON versus REV: *p <* 10^−4^, LOS versus REV: *p =* 0.6317. The center line indicates the median, the box represents the 25th–75th percentiles, and the whiskers show the minimum and maximum values (n = 6 rats). E–G) Difference in normalized numbers of pairwise coactivation of place cells in templates corresponding to the changed reward side and unchanged reward side during PBEs in Rest 1 session (E), Rest 2 session (F), and NREM sleep (G). One‐way ANOVA with Tukey post hoc analysis, Rest 1 session: F (2, 15) = 0.4202, *p =* 0.6644, CON versus LOS: *p =* 0.7695, CON versus REV: *p =* 0.9844, LOS versus REV: *p =* 0.6698; Rest 2 session: F (2, 15) = 0.7515, *p =* 0.4886, CON versus LOS: *p =* 0.4586, CON versus REV: *p =* 0.7733, LOS versus REV: *p =* 0.8562; NREM sleep: F (2, 15) = 7.046, *p =* 0.0070, CON versus LOS: *p =* 0.0071, CON versus REV: *p =* 0.0383, LOS versus REV: *p =* 0.6753. The center line indicates the median, the box represents the 25th–75th percentiles, and the whiskers show the minimum and maximum values (n = 6 rats). ***p < 0.001; **p < 0.01; *p < 0.05; ns, *p >* 0.05.

To examine place cell activity with greater precision, we further analyzed replay events within PBE events. Within some PBEs, the spike sequences of activated place cells replayed the sequences during running, in either forward or reverse order.^[^ ^10^, ^33^, ^34^ ^]^ To analyze these events, we performed Bayesian decoding of PBEs using a template‐based method described in previous studies.^[^ ^25^, ^33^, ^35^, ^36^, ^37^ ^]^ A PBE event with a shuffled p value less than 0.05 corresponding to any place cell template was defined as a replay event. PBEs with spike sequences displaying the replay of a template sequence in either forward or reverse order were identified by Bayesian decoding and linear regression (**Figure** **4**A).

We investigated the replays in all offline sessions, including Rest 1, Rest 2, and Sleep sessions. We computed the replay rates during Rest 1, Rest 2, and NREM sleep. The result showed no significant change in replay rates among CON, LOS, and REV periods during Rest 1, Rest 2, and NREM sleep (Figure S7A–C, Supporting Information). Based on the decoded templates of each replay, we categorized replays as left‐runs (including left outbound and left inbound templates) or right‐runs (including right outbound and right inbound templates) and computed regional preferences for the changed reward side.

The regional preferences of replays during the Rest 1 session and Rest 2 session showed no significant change across the three memory updating periods (Figure 4B,C). However, regional preferences for the novel, larger reward side during NREM sleep were significantly increased in the LOS period compared to the CON period. Regional preferences for the changed reward side did not continue to rise significantly in the REV period compared to the LOS period (Figure 4D). The regional distribution of template cells was consistent across the three periods (Figure S4G,H, Supporting Information), which ruled out the bias of decoded replay regions caused by the bias of template place cell distributions. These results suggested that the regional distribution of replays during awake rest was not significantly linked to either changes in reward magnitude or the shifts in preference indicating memory updating. However, the regional distribution of replays during NREM sleep changed in line with the new reward ratio in the LOS periods.

Previous studies showed that the presence of rewards may cause replays to point toward the reward regions.^[^ ^25^ ^]^ To perform a more detailed analysis of replay distributions, we constructed replay vectors for sleep replays, indicating the trajectory from the decoded start position to the decoded end position (Figure S6E, Supporting Information). Among replays corresponding to the changed reward side, we calculated the proportion of replays pointing toward the reward site (i.e., forward replays for outbound template replays and reverse replays for inbound template replays). These ratios in the two awake rest sessions and NREM sleep remained unchanged throughout the memory updating process (Figure S6F–H, Supporting Information). These results indicated that although replays during NREM sleep exhibited a significant increase in regional preference to the new and larger reward in both the LOS and REV periods, the ratio of replays pointing toward the new and larger reward did not show a significant change. In addition to analyzing the proportion of replays pointing toward the reward site, we also examined the directional distribution of replays (forward and reverse) corresponding to different place cell templates. The results showed that, although during the LOS and REV periods the replays in NREM sleep exhibited a significantly increased preference for the novel, larger reward side compared to the CON period, the proportion of forward replays within the replays of each template did not show any significant change (Figure S6I–L, Supporting Information).

Given that Bayesian decoding for replay analysis is sensitive to the number of simultaneously recorded neurons and may introduce biases when the number of neurons is small, we complemented this approach with an analysis of pairwise coactivation in template place cells, a method less sensitive to neuron population size.^[^ ^38^ ^]^ Specifically, we calculated the differences in normalized numbers of pairwise coactivation of place cell templates corresponding to the changed reward side and unchanged reward sides during PBEs in the Rest 1, Rest 2, and Sleep sessions. This metric reflected the bias in place cell pair coactivation toward the changed reward side. Our results revealed that, in the Rest 1 and Rest 2 sessions, the difference in normalized pairwise coactivation count showed no significant changes throughout the memory updating process. In contrast, during PBEs in NREM sleep, this value was significantly elevated in the LOS period and exhibited a slight, statistically non‐significant decrease in the REV period compared to the LOS period (Figure 4E–G). These results about template place cell pair coactivation were consistent with and reinforced the results obtained from replay analysis using the Bayesian decoding method. These results indicated that increases in reward magnitude made the pairwise coactivations and replays of place cells during NREM sleep following online behaviors biased toward the region corresponding to the increased reward, while the orientations of the replays were not significantly changed.

Taken together, our analyses of place cell activities during Rest 1, Rest 2, and Sleep sessions suggested that hippocampal activity during NREM sleep was more strongly associated with memory updating than activity during awake rest. These results were consistent with previous studies showing that SWRs and replay during awake rest were not necessary for either short‐term or long term memory performance,^[^ ^39^ ^]^ whereas SWRs during sleep played a significant role in long‐term memory performance.^[^ ^14^ ^]^ These findings further suggested that memory updating and consolidation may share similar underlying mechanisms.

### 2.5 Replays During Reward Consumption Predict Future Choices

**Figure 5:**
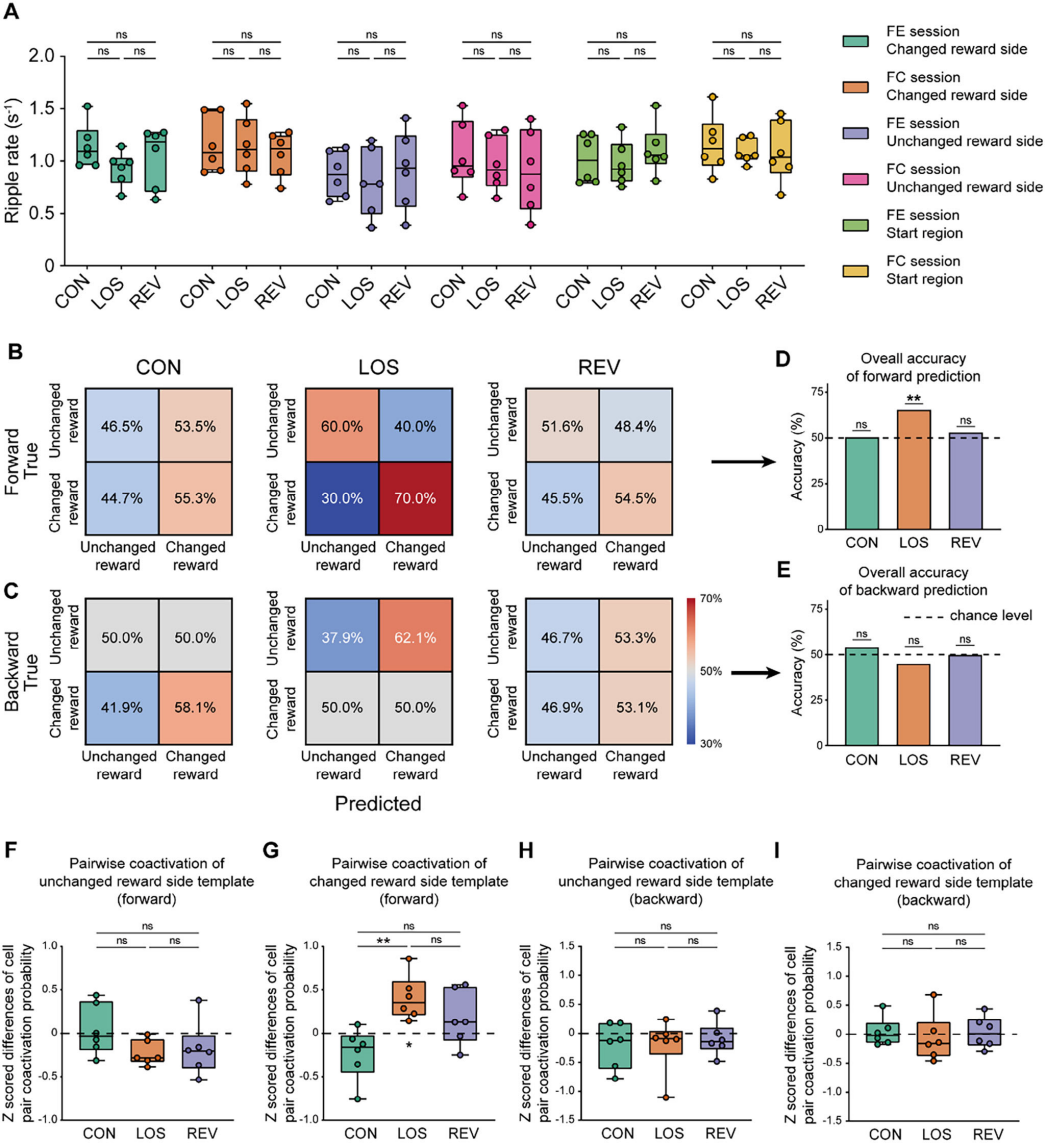
Replays during reward consumption predict future choices. A) Ripple rates during reward consumption in the changed reward site, unchanged reward site, and Start region in the Forced Entry (FE) session and Free Choice (FC) session. Data were organized into six independent conditions, each containing three subgroups (CON, LOS, and REV periods). Within each experimental condition, a one‐way ANOVA with Tukey post hoc analysis was performed to assess differences among the three subgroups. Changed reward side in FE session: F (2, 15) = 1.347, *p =* 0.2898, CON versus LOS, *p =* 0.2602, CON versus REV, *p =* 0.7318, LOS versus REV, *p =* 0.6613; changed reward side in FC session: F (2, 15) = 0.2248, *p =* 0.8013, CON versus LOS, *p =* 0.9919, CON versus REV, *p =* 0.8051, LOS versus REV, *p =* 0.8676; unchanged reward side in FE session: F (2, 15) = 0.2131, *p =* 0.8105, CON versus LOS, *p =* 0.9042, CON versus REV, *p =* 0.9756, LOS versus REV, *p =* 0.8004; unchanged reward side in FC session: F (2, 15) = 0.3371, *p =* 0.7191, CON versus LOS, *p =* 0.8926, CON versus REV, *p =* 0.6972, LOS versus REV, *p =* 0.9300; start region in FE session: F (2, 15) = 0.5200, *p =* 0.6048, CON versus LOS, *p =* 0.9383, CON versus REV, *p =* 0.7885, LOS versus REV, *p =* 0.5864; start region in FC session: F (2, 15) = 0.1603, *p =* 0.8533, CON versus LOS, *p =* 0.8839, CON versus REV, *p =* 0.8704, LOS versus REV, *p =* 0.9995. The center line indicates the median, the box represents the 25th–75th percentiles, and the whiskers show the minimum and maximum values (n = 6 rats for each subgroup). B,C) Confusion matrix of predictions. Upper row (B): accuracy between prediction based on replay distribution and next choice; lower row (C): accuracy between prediction and previous choice; columns from left to right: CON, LOS, and REV periods. D) Accuracy of forward predictions in CON, LOS, and REV periods. One‐sided binomial test for each condition, CON period: accuracy = 50.6%, *p =* 0.5000, n = 81 trials; LOS period: accuracy = 65.7%, *p =* 0.0057, n = 70 trials; REV period: accuracy = 53.1%, *p =* 0.3540, n = 64 trials. Bar graphs represent accuracy values without error bars. E) Accuracy of backward predictions in CON, LOS, and REV periods. One‐sided binomial test for each condition, CON period: accuracy = 54.3%, *p =* 0.2526, n = 81 trials; LOS period: accuracy = 45.1%, *p =* 0.4767, n = 71 trials; REV period: accuracy = 50.0%, *p =* 1.000, n = 62 trials. Bar graphs represent accuracy values without error bars. F) Z‐scores of the differences in pairwise coactivation probabilities of unchanged reward side template place cell pairs between reward consumption following different choices in the Free Choice session. One‐way ANOVA with Tukey post hoc analysis: F (2, 15) = 1.830, *p =* 0.1945; CON versus LOS: *p =* 0.2070; CON versus REV: *p =* 0.3268; LOS versus REV: *p =* 0.9499. n = 6 rats. G) Z‐scores of the differences in pairwise coactivation probabilities of changed reward side template place cell pairs between reward consumption following different choices in the Free Choice session. One‐way ANOVA with Tukey post hoc analysis: F (2, 15) = 7.415, *p =* 0.0058; CON versus LOS: *p =* 0.0046; CON versus REV: *p =* 0.0681; LOS versus REV: *p =* 0.3810. One sample t‐test, CON versus 0: *p =* 0.1206; LOS versus 0: *p =* 0.0111; REV versus 0: *p =* 0.2167. n = 6 rats. H) Z‐scores of the differences in pairwise coactivation probabilities of unchanged reward side template place cell pairs between reward consumption followed by different choices in the Free Choice session. One‐way ANOVA with Tukey post hoc analysis: F (2, 15) = 0.1476, *p =* 0.8641; CON versus LOS: *p =* 0.9994; CON versus REV: *p =* 0.8788; LOS versus REV: *p =* 0.8939. n = 6 rats. I) Z‐scores of the differences in pairwise coactivation probabilities of changed reward side template place cell pairs between reward consumption followed by different choices in the Free Choice session. One‐way ANOVA with Tukey post hoc analysis: F (2, 15) = 0.2076, *p =* 0.8149; CON versus LOS: *p =* 0.8358; CON versus REV: *p =* 0.9994; LOS versus REV: *p =* 0.8529. n = 6 rats. The center line indicates the median, the box represents the 25th–75th percentiles, and the whiskers show the minimum and maximum values (F‐I). ***p < 0.001; **p < 0.01; *p < 0.05; ns, *p >* 0.05.

Previous studies have demonstrated that changes in reward sizes can influence SWRs and replays during reward consumption in the maze.^[^ ^40^ ^]^ Therefore, we examined whether memory updating affected SWRs and replays during online reward consumption. First, we analyzed ripples during reward consumption at the two reward sites (in the changed reward side and unchanged reward side) and in the start region during the two online sessions. The results showed that ripple rates during reward consumption across all three regions and both online sessions remained unchanged throughout the memory updating process (**Figure** **5**A). Therefore, in contrast to previous studies on short‐term memory, ripple rates during online reward consumption remained unchanged during the updating of long‐term spatial associative memory.

Considering that reward consumption in the start region, unlike that in the reward sites on the horizontal arm, linked the previous trial to the next one. Thus, we investigated whether the neural activities during reward consumption in the start region were involved in memory updating. Ripple length has been shown to positively correlate with memory demands in memory‐related behavior.^[^ ^41^ ^]^ Therefore, we measured the duration of ripples during reward consumption in the start region between consecutive trials in the running sessions, including both the Forced Entry session and Free Choice session. Our results indicated that, throughout the memory updating process, there were no significant differences in ripple length between consecutive trials in either running session (Figure S8A,B, Supporting Information). Then, we investigated whether replays during this time range were associated with the decisions in the maze as described in previous studies.^[^ ^38^, ^42^, ^43^, ^44^ ^]^ We conducted a trial‐by‐trial analysis to determine whether the regional preference of replays aligned with either the previous or subsequent trial during the Free Choice session. We found that the regional preferences of replays during reward consumption in the Start region significantly predicted future choices in subsequent trials only in the LOS period but not in the CON or REV period (Figure 5B,D; Figure S8C, Supporting Information). However, the regional preferences of replays during reward consumption in the Start region were not affected by the previous trials in any of the three periods (Figure 5C,E). In contrast to previous studies on working memory, which suggested that replay primarily reflects past experiences,^[^ ^45^ ^]^ our results showed that during memory updating, replays between trials more strongly reflected the animal's future choices during the LOS period.

Previous studies have shown that pairwise coactivation of place cells during PBEs predicts correct and incorrect behavioral choices, with this effect being particularly pronounced during initial learning.^[^ ^38^ ^]^ Therefore, we investigated the pairwise coactivation of place cells during reward consumption between trials in the Free Choice sessions. To investigate this, we assigned reward consumption in the start region to the subsequent trial and calculated the z‐score of the difference in pairwise coactivation probability of template place cell pairs of both sides between PBEs associated with ipsilateral and contralateral trials. Our results revealed that, for the template of changed reward side, the z‐score of template cell pairwise coactivation was significantly greater than zero during the LOS period and significantly higher than in the CON period. In contrast, the z‐score in the REV period was not significantly greater than zero and showed no significant difference compared to the CON period (Figure 5G). For the unchanged reward side, the z‐score remained not significantly greater than zero and exhibited no significant changes throughout the memory updating process (Figure 5F). These findings indicated that only during the LOS period did the pairwise coactivation of template cell pairs on the changed reward side significantly predict the animal's behavioral choices.

To further explore whether template place cell pair coactivation in PBEs during reward consumption in the start region reflects the behavioral choice of the previous trial, we assigned reward consumption to the preceding trial and performed the same z‐score computation. The results showed that, for both the changed reward side and unchanged reward side template cell pairs, the z‐scores did not significantly change across the memory updating process and were not significantly greater than zero at any period (Figure 5H,I). These results aligned closely with findings from forward and backward prediction analyses using Bayesian decoding of replay. Collectively, these results demonstrated that, during memory updating, only in the LOS period did neuronal activity in PBEs predict future behavioral choices, with this predictive capacity primarily driven by the place cell template associated with the changed reward side.

### 2.6 Both Online and Offline Replays on the Non‐Updating Days Stay Unchanged

In the behavioral data, four rats began to exhibit a clear preference for the side associated with the new and larger reward on the third day of Stage 3 (Figure 1C; Figure S1A, Supporting Information). This indicated that the LOS periods for these animals occurred on the second day of Stage 3. On the first day of Stage 3, although the rats experienced changed reward amount ratios, memory updating did not occur, as no preference reversal was observed on the following day. The above analyses revealed a correlation between hippocampal activity during the LOS period and subsequent reversal of behavioral preference. To further assess the specificity of this relationship, we defined the first day of Stage 3 in these four rats as a non‐updating (NU) period and analyzed hippocampal activity during this phase.

**Figure 6:**
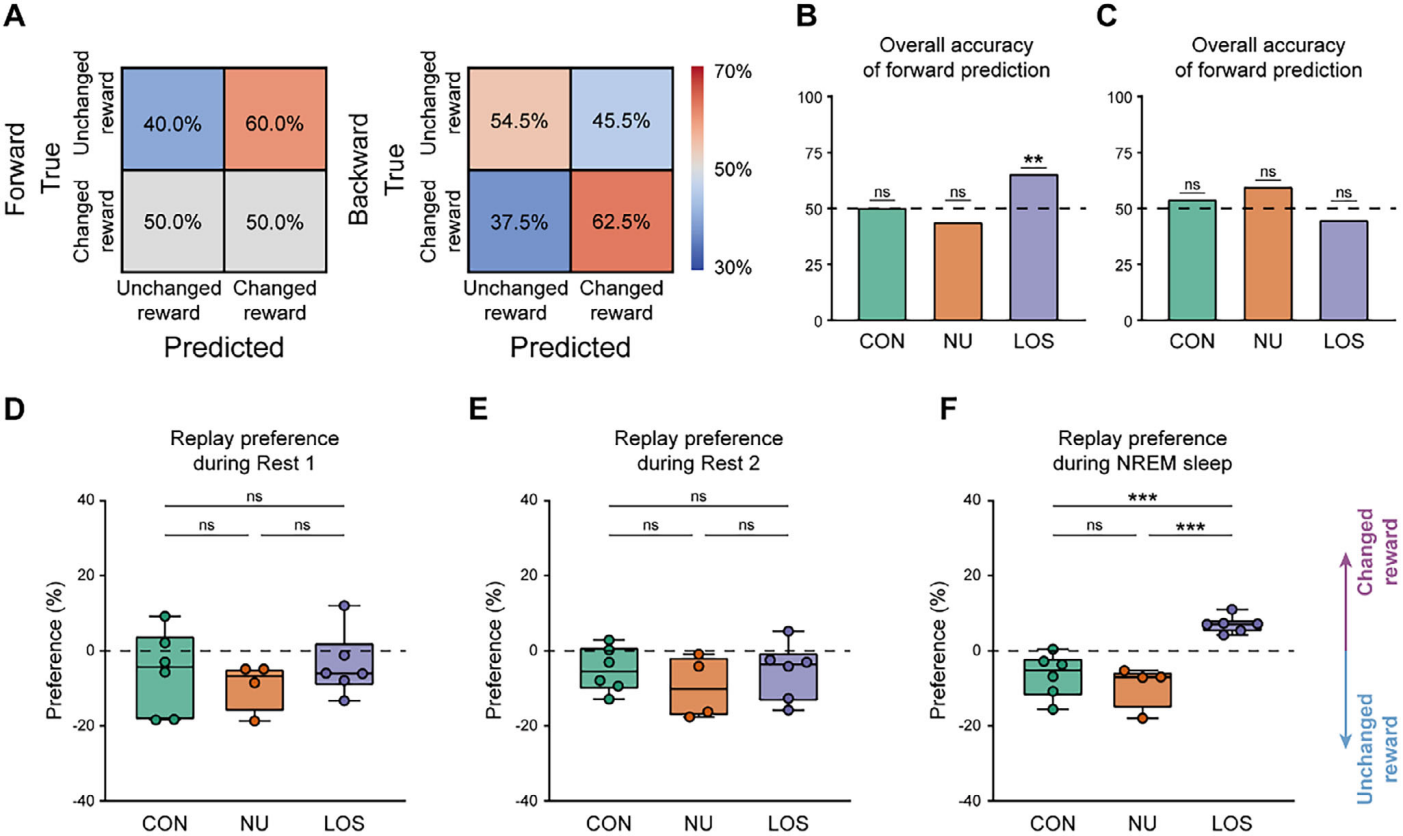
Replays on the non‐updating days stay unchanged. A) Confusion matrix of predictions in the NU periods. Left: accuracy between prediction based on replay distributions and next choices; right: accuracy between prediction and previous choice. B) Accuracy of forward predictions in CON, NU, and LOS periods. The data of CON & LOS periods are the same as in Figure 5D. One‐sided binomial test for NU period: accuracy = 44.1%, *p =* 0.6076, n = 34 trials. Bar graphs represent accuracy values without error bars. C) Accuracy of backward predictions in CON, NU, and LOS periods. The data of CON & LOS periods are the same as in Figure 5D. One‐sided binomial test for NU period: accuracy = 60.0%, *p =* 0.3105, n = 35 trials. Bar graphs represent accuracy values without error bars. D–F) Regional preferences of replays during Rest 1 session (D), Rest 2 session (E), Sleep session (F) in the LOS, NU, and LOS periods. The data for CON & LOS periods are the same as Figure 4B–D. One‐way ANOVA with Tukey post hoc analysis, Rest 1 session: F (2, 13) = 0.4285, *p =* 0.6604, CON versus LOS: *p =* 0.8239, CON versus REV: *p =* 0.9295, LOS versus REV: *p =* 0.6353; Rest 2 session: F (2, 13) = 0.5755, *p =* 0.5761, CON versus LOS: *p =* 0.5872, CON versus REV: *p =* 0.9932, LOS versus REV: *p =* 0.6470; Sleep session: F (2, 13) = 17.92, *p =* 2 × 10^−4^, CON versus LOS: *p =* 0.6485, CON versus REV: *p =* 8 × 10^−4^, LOS versus REV: *p =* 4 × 10^−4^. The center line indicates the median, the box represents the 25th–75th percentiles, and the whiskers show the minimum and maximum values (n_CON_ = 6 rats, n_NU_ = 4 rats, n_LOS_ = 6 rats). ***p < 0.001; **p < 0.01; *p < 0.05; ns, *p >* 0.05.

We first conducted a trial‐by‐trial prediction analysis. The results revealed that, in contrast to the LOS period, hippocampal replay events occurring during reward consumption in the Start region during the NU period did not predict either subsequent choices or preceding experiences (**Figure** **6**A–C). These findings suggested that the predictive power of online hippocampal replays for future behavior was specifically correlated with the initiation of memory updating.

Next, we analyzed offline replays, including those during awake rests and sleep sessions. The results showed that in all three offline sessions, the PBE rate (Figure S9A–C, Supporting Information) and replay rate (Figure S9D–F, Supporting Information) during NU periods were not significantly different from those during the CON and LOS periods. For the two awake rest sessions, the regional preferences for the changed reward side were unchanged across the CON, NU, and LOS periods (Figure 6D,E). For the Sleep session, the regional preferences of the novel larger reward side during the NU period were also not significantly different from those in the CON period and significantly lower than during the LOS period (Figure 6F). These results indicated that the content of sleep replay remained unchanged before the memory updating was initiated.

These results suggested that, despite changes in reward magnitude ratios during the NU period, the neural activity in this period resembled that of the CON period more closely than the LOS period. Consequently, these findings further strengthened the specific association between place cell activity in the LOS period and memory updating.

### 2.7 Discussion

In this study, we investigated how hippocampal place cell replays during different behavioral states are involved in the process of long‐term spatial associative memory updating. Our findings highlighted the critical role of the hippocampus in memory updating and deepened our understanding of the functional differences of hippocampal replays across different behavioral states.

#### 2.7.1 A T‐Maze‐Based Behavioral Paradigm Describes Memory Updating

We introduced a behavioral paradigm that reveals long‐term memory formation and updating in rats, wherein the animals exhibited a long‐term preference for one side of a maze based on a fixed reward ratio. When the reward amount on one side is altered, the rats’ memory contents should be updated in accordance with this new reward magnitude ratio. Our findings indicated that this updating process in this paradigm was typically a two‐period process (LOS period and REV period). To investigate whether CA1 place cells play a role in this process, we analyzed the activity of place cells during several states: running in the maze, reward consumption in the maze, awake rest, and NREM sleep. Our results revealed the following during memory updating: (1) theta power during running in the maze stayed unchanged, (2) the predictive power of replays during reward consumption at the Start region significantly increased only in the LOS period, (3) SWRs and replays during awake rests in the rest box were unchanged, (4) the regional preferences of replays corresponding to the changed reward side increased in the LOS and REV periods, although the detailed distribution of these replays stayed unchanged, (5) the elevated predictive power of replays during reward consumption and replay regional preferences toward the side with increased reward during NREM sleep did not emerge during the period in which the rewards have been changed while the memory updating does not start.

Although the T‐maze is typically used to assess hippocampus‐dependent working memory,^[^ ^46^ ^]^ we employed it here for a long‐term memory task. Similar to the performance of rats seeking food in the fixed three arms of an eight‐arm radial maze,^[^ ^14^, ^39^ ^]^ our rats gradually developed a preference for the side with the larger reward in the T‐maze (Figure 1D). It is noteworthy that a few rats exhibited weak preferences for the 4‐s reward during this stage, which indicated that the difference between the 4‐s and 2‐s rewards may not be salient for every rat. Nevertheless, this phenomenon had no effect on the occurrence of memory updating after the change of reward magnitude ratio in the paradigm. Moreover, we found that memory updating in our paradigm did not take place immediately after the reward ratio change. Across all animals, there was consistently a short period with no preference between the two sides (LOS period), followed by a reversed preference (REV period). It is noteworthy that in the Free Choice sessions of the LOS period, the real‐time preferences of the rats stayed steady across trials (Figure S2A–H, Supporting Information). Since the animals’ preference for the new and larger reward did not show a further significant increase in the following days compared to the REV period, this suggests that the main process of memory updating was largely completed before the REV period. Consequently, the primary process of memory updating was likely to occur during the LOS period. These findings indicated that our behavioral task provided a framework for studying the updating of long‐term spatial associative memory.

Prediction errors are the primary factor driving long‐term memory updating.^[^ ^47^, ^48^ ^]^ It refers to the discrepancy between real‐world experiences and memory‐based expectations when the environment changes, such as alterations in unconditioned stimulus,^[^ ^3^ ^]^ changes of context,^[^ ^49^ ^]^ or shifts in timing.^[^ ^50^, ^51^ ^]^ The presence of prediction error causes updating of the content of long‐term memories that have already consolidated, distinguishing it from the entirely new learning processes driven by novelty.^[^ ^52^, ^53^ ^]^ In this study, changes in reward magnitude were introduced solely by increasing the reward amount. Therefore, investigating how different types of prediction errors affect memory updating would be a valuable direction for future research, as it could help to clarify whether changes in replay content simply reflect the prediction errors or the content of new experiences.

According to previous studies,^[^ ^3^, ^54^, ^55^ ^]^ memory updating consists of two processes: destabilization, during which consolidated long‐term memory becomes labile again, and reconsolidation, during which new experiences are integrated into the destabilized memory. Destabilization occurs upon memory retrieval, while reconsolidation primarily takes place during the subsequent offline period, such as rest and sleep, within a time window of several hours.^[^ ^56^ ^]^ Given that memory updating in our paradigm primarily occurred during the LOS period, we inferred that destabilization took place during the online sessions (including the Forced Entry and Free Choice sessions), whereas reconsolidation occurred during the subsequent offline states (including the Rest 1, Rest 2, and Sleep sessions). Future research at the level of gene expression and synaptic plasticity will help us better understand the cellular mechanisms underlying these two physiological processes.

#### 2.7.2 Changes in Replays Occur Only in One Type of Offline State

In the behavioral paradigm introduced in this study, animals were required to perform different tasks during the Forced Entry and Free Choice sessions. To examine whether this task transition influenced the spatial firing patterns of hippocampal place cells, we computed the Pearson correlation between the 1D firing fields of place cells across the two sessions. The results showed that the majority of place cells maintained stable firing fields between the two running sessions on the same day. The stability of place fields across the Forced Entry (FE) and Free Choice (FC) sessions for most cells allowed us to use the combined firing rate distributions derived from both the Forced Entry and Free Choice sessions for subsequent analyses, without the need to construct separate templates for the FC and FE sessions.

In this study, we investigated the activities of hippocampal place cells within PBEs at three distinct levels. Analysis of place cell activation provided a population‐level measure of how spatial environments were represented across hippocampal neuronal ensembles.^[^ ^25^, ^57^, ^58^ ^]^ In contrast, Bayesian decoding of replay events enabled the reconstruction of temporally ordered sequences of place cell activity, thereby allowing inference of specific trajectory information embedded within these events.^[^ ^20^, ^33^, ^35^ ^]^ Additionally, we examined pairwise coactivation of place cells to assess the statistical dependencies between neuronal firing patterns.^[^ ^38^, ^59^ ^]^ Compared to Bayesian decoding, pairwise coactivation analysis is less sensitive to the total number of simultaneously recorded neurons and exhibits reduced susceptibility to sampling bias when cell counts are limited. Accordingly, pairwise coactivation was employed as a complementary approach to Bayesian decoding in the analysis of replay events. Notably, the outcomes from all three analytic methods were broadly consistent, thereby reinforcing the robustness and reliability of our findings.

Furthermore, previous studies have shown that assembly reactivation of place cells encodes discrete environmental states, whereas sequential replay supports associative memory.^[^ ^60^ ^]^ Although our study primarily focused on the analysis of sequential replay, the consistency between the results of replay and pairwise coactivation patterns suggested that assembly reactivation of place cells may also exhibit similar changes during memory updating.

Previous studies have shown that the reconsolidation phase of memory updating typically occurs during offline states, including rest and sleep.^[^ ^3^, ^61^ ^]^ Our results showed that there was no significant difference in ripple rates and PBE rates during the two rest sessions across the memory updating process. Knowing that hippocampal place cells are activated during the PBEs, we analyzed the regional preferences of place cells activated within PBEs in the two rest sessions. Regional preferences of place cell activation during the two rest sessions in the LOS periods were not significantly changed compared to those in the CON periods. The regional preferences of replays and the difference between the pairwise coactivations of template place cells during rest sessions were consistent with this result. Although the regional preference of activations toward the increased reward side increased in the REV period, the analysis of replays and pairwise coactivations did not show a similar result. These findings raised the possibility that this increased activation did not appear to be organized into temporally structured coactivation or sequential replay events.

Now that replays during awake rests exhibited no significant change during the memory updating process, we next focused on another offline state, sleep, which is also highly related to memory reconsolidation. Regarding Sleep sessions, given that ripples and replays predominantly occur during NREM sleep,^[^ ^62^ ^]^ we analyzed time epochs corresponding to NREM sleep only. Our results showed that although PBE rates and replay rates during NREM sleep remained stable throughout the memory updating process, the regional preferences of both place cell activations and replays were significantly biased toward the reward‐increasing side, and slightly further increased in the REV periods. Further analysis of the detailed distribution revealed that although the replays corresponding to the changed reward side increased, the tracks of these replays did not converge to the reward position. These results suggested that the regional ratio of replays during sleep reflected the relative size of the rewards, and this ratio functioned as the content written during memory reconsolidation.

During NREM sleep, a triple coupling among cortical slow oscillations (SO), thalamic spindles, and hippocampal ripples has been identified as a key mechanism underlying system consolidation of memory.^[^ ^63^, ^64^, ^65^ ^]^ Our results showed that changes in hippocampal replay occurred specifically during sleep, rather than during awake rest, which supports the idea that sleep‐specific network mechanisms—such as this triple coupling—play an important role in memory updating.

#### 2.7.3 Hippocampal Replays Predict Animals’ Behaviors During Memory Updating

Although the theta oscillations are well‐known to play an essential role in short‐term memory encoding,^[^ ^66^, ^67^ ^]^ our results demonstrated that theta‐band power during running in the maze remained stable throughout the process of long‐term memory updating. In addition to running, the rats also consumed rewards in the maze, which was a period characterized by ripples and replays. Our findings showed that ripple rates on both the changed reward side and in the Start region remained unchanged during memory updating. However, the regional distribution ratio of replays during reward consumption in the Start region exhibited a significant increase in predictive power regarding the rats’ next move during the Free Choice session only in the LOS period. This result suggested that in the LOS period, the rats may deliberate on their next move between trials. According to the system consolidation theory,^[^ ^68^, ^69^ ^]^ long‐term memory becomes less dependent on the hippocampus and more reliant on the prefrontal cortex. Our results suggested that when memory updating occurs, the hippocampus may temporarily regain the dominant role.

Prior research has shown that pairwise coactivation of place cells exhibits predictive power in behavior, and this predictive power is confined to the initial learning phase.^[^ ^38^ ^]^ Consistent with this, our findings showed that the z‐scored differences in pairwise coactivation probability for template place cells on the changed reward side, indicative of predictive power, were significantly greater than zero only during the LOS period, but not during the REV period. These results suggested that in the long‐term memory paradigm used in this research, the memory updating induced by the change of reward magnitude (introduction of prediction error) returned the stabilized long‐term memory to a state akin to initial learning in the LOS period.

#### 2.7.4 Changes in Replays do not Occur before the Initiation of Memory Updating

We noticed that before the memory updating happens, there may be one day in which the rats showed a preference consistent with the CON period or no preference. Because these days were not followed by the REV period, we believed that the memory updating does not occur mainly in these days. The key criterion for distinguishing between the LOS period and the NU period was whether they immediately precede the REV period. After the REV period, the animals' behavior entered a relatively stable stage where their preferences for the new and larger reward no longer showed a significant further increase. Therefore, we concluded that memory updating primarily occurred on the day immediately before the REV period (i.e., the LOS period). In contrast, the day preceding the LOS period in stage 3 (if it existed) must differ functionally from the LOS period, as it did not directly lead to the formation of a clear preference for the new and larger reward ‐ hence we designated this as the NU period.

Our analysis showed that both online and offline replays of the NU period showed similar properties to the CON period. Online replays in the Start region during the NU period could not predict the animals’ choice in the following trial of the Free Choice session. And replays during NREM sleep did not bias toward the increased reward side. These results of the NU period emphasized the specificity of the relationship between the changes of both online and offline replays and the day immediately before the preferences were reversed (LOS period).

Given that these specific changes in both online and offline replays accompanied the long‐term memory updating initiated by changes of reward magnitude, it remains to be determined how reward prediction error signals interact with the hippocampus to induce such changes, and what factors determine the timing of memory updating. Future studies investigating the interactions between the hippocampus and the dopaminergic system may provide further insights into the mechanisms underlying memory updating.

## 3 Conclusion

In summary, this study introduces a behavioral paradigm and identifies potential hippocampal mechanisms underlying memory updating. The memory updating process spans three periods: CON period, LOS period, and REV period, with the LOS period serving as the primary window during which memory updating occurred. The replays of place cells during behavior exhibit significant predictive power with respect to subsequent behavioral choices exclusively in the LOS period. Furthermore, the regional preferences of replays during NREM sleep in the LOS period are significantly biased toward the spatial location associated with the new and larger reward. These phenomena are specific to the LOS period while notably absent before the onset of memory updating, underscoring the temporal specificity of the observed hippocampal dynamics.

## 4 Experimental Section

### Subjects

Adult male Long‐Evans rats, aged 12 to 16 weeks, weighing between 400 and 600 grams, were obtained from Charles River Laboratories (Substrain: Crl:LE; RRID: RGD_2 308 852). The animals were housed individually in single cages, maintained at a controlled room temperature of 23 °C, and kept on a 12‐h light‐dark cycle. Food and water were available without restriction, except during the experimental periods requiring water restriction. All experiments were conducted in accordance with the guidelines of the Animal Care and Use Committee of the Peking University Health Science Center (DLASBD0099).

### Behavioral Apparatus

The behavioral apparatuses used in this study comprise a continuous T‐maze, a rest box, and a sleep box. The continuous T‐maze consists of a 68 cm central arm, an 84 cm horizontal arm, and two 78 cm side arms. The width of the arms is 15 cm. The maze is equipped with three nose‐poke devices, one in the Start region and two at the terminals of the horizontal arm. Upon nose poking, sucrose water is delivered in a consistent flow velocity (≈30 µL s^−1^) for 2 (small reward), 4 (medium reward), or 8 s (large reward). There is also an infrared beam detector positioned on the central arm to automatically detect the entry of the rats.

The rest box is an elevated flowerpot tray located within an enclosed box (Figure S3B, Supporting Information). In the rest box, the rat's movement was confined to the small area of the flowerpot tray, preventing free locomotion. Therefore, the animal was considered to be in an immobile state throughout the Rest 1 and Rest 2 sessions. Starting from the beginning of animal handling and throughout the experiment (except during the postoperative recovery period), rats underwent at least two 20‐min habituation sessions in the rest box each day. This ensured that the animals were well adapted to the rest box environment and maintained a relatively quiet but awake state during recordings.

The sleep box is an acrylic cylinder with a diameter of 40 cm and a height of 40 cm. A hole is drilled in the cover to allow the cable to pass through. The floor is covered with bedding substrate from its home cage. The sleep box was located in the same room as the continuous T‐maze. Throughout the behavioral paradigm, including the pre‐training stage, rats were housed in the sleep box to ensure familiarity with the environment, thereby facilitating the occurrence of natural sleep during recordings. This design minimized the potential influence of environmental novelty on sleep patterns and neural activity, allowing for reliable recordings during sleep sessions.

### Behavioral Task

The rats were handled for 1 week before the initiation of the training procedures. Two days before the training began, the animals’ daily water consumption was restricted, maintaining their weights no less than 85% of the baseline level. Food was ad libitum throughout the entire experiment. The training protocol comprised two phases:

### Phase 1: Pre‐Training

In this phase, prior to surgery, the rat was trained with 1 to 2 Forced Entry sessions per day. During each trial, the rat was placed in the Start region of the T‐maze. After poking at the water port in the Start region and receiving a sucrose water reward (10% sucrose solution delivered via a water pump for 4 s, ≈130 µL), an auditory cue signaled the onset of a new trial. The rat was guided by an illuminated light at the end of the central arm to run along the central arm. Upon triggering an infrared detector in the central arm, a light at one of the two terminals in the horizontal arm illuminated, directing the rat toward the corresponding terminal for a reward (also a 10% sucrose solution for 4 s). A subsequent light cue in the Start region, coupled with another auditory cue, prompted the rat to return to the Start region to receive a second sucrose water reward and initiate the next trial. The rewarded sides were chosen pseudo‐randomly. Each Forced Entry session during pre‐training consisted of 50 trials or lasted 60 min. The criterion for this phase was consecutively finishing 50 trials within 50 min on two consecutive days. After meeting this criterion, the rat was provided with ad libitum access to water and food for two days before undergoing surgery.

### Phase 2: Post‐Training. (Stage 1 of the Behavioral Paradigm)

Following recovery from surgery, the rat was trained with a Forced Entry session and a Free Choice session before electrophysiological recording. The Forced Entry session followed the same protocol as during pre‐training, but lasted either 30 trials or 30 min, whichever was reached first. Following the Forced Entry session, the rat was placed in the rest box for 20 min before commencing the Free Choice session. During the Free Choice session, once the infrared detector in the central arm was triggered, lights at both terminals of the horizontal arm were illuminated, allowing the rat to freely choose between the two terminals. In both the Forced Entry and the Free Choice sessions, there was no additional delay between trials. This session lasted either 20 trials or 30 min, whichever came first. Following the Free Choice session, the rat was placed in the rest box for another 20 min. The criterion for this phase was the successful completion of 30 Forced Entry trials within 30 min and 20 Free Choice trials within 20 min for five consecutive days. Upon meeting these criteria, the rat was prepared for electrophysiological recording.

### Surgery

Custom microdrives with 16 independently movable tetrodes were implanted above the right or bilateral hippocampi (AP: −3.8 mm, ML: ±2.4 mm, DV: −1.0 mm). Bone screws and a protective shell were placed in the skull. The bone screws, the base of the microdrive, and the base of the shell were covered with dental cement to fix the microdrive. Three bone screws were connected to the ground (GND) wire of the recording system. The inner surface of the protective shell was covered with copper tapes and connected to the GND wire to form a Faraday cage. A red LED for animal head tracking was soldered onto the headstage.

The surgery was performed under isoflurane anesthesia. For induction of anesthesia, 3% isoflurane was used, and for maintenance of anesthesia, 1.0 to 1.5% isoflurane was used. After surgery, the rats were singly housed and allowed to recover for at least a week with ad libitum access to water and food.

### Recording Procedure

Over a 2–3 week postoperative period, tetrodes were slowly advanced into the CA1 region until distinguishable SWRs appeared. The recording procedure started after the tetrodes had not been moved for at least 24 h.

1 week after the surgery, water restrictions were resumed. And the rats performed post‐training to make them habituate with the electrode and recording cable. During the training stage, rewards at left and right reward sites were both 4 s (≈130 µL).

After the rats could complete the Forced Entry session and Free Choice session within 30 and 20 min, respectively, for 5 consecutive days. The recording procedure started. The recording procedure consisted of a 5‐day 4 s versus 2 s block (stage 2) and a 5‐day 4 s versus 8 s block (stage 3). For animals exhibiting unequal side preferences in stage 1 (post‐training stage), the side with the higher preference was designated as the changed reward side, in order to prevent intrinsic bias from confounding the preference assessment in stage 2. On every recording day, the rats performed a Forced Entry session, a Rest 1 session, a Free Choice session, a Rest 2 session, and were placed back in the sleep box for an ≈2‐h Sleep session. During sleep recording in the sleep box, animals were temporarily given free access to water to ensure that their natural sleep was not affected by water restriction. In vivo electrophysiological data were recorded throughout the whole procedure.

### Data Acquisition

In vivo electrophysiological recordings were performed using unilateral or bilateral electrodes. An electrode consists of 16 tetrodes made of tungsten wires (20 µm in diameter, California Fine Wires Company) and a custom 3D printed unilateral or bilateral microdrive. The impedance of each channel was between 500 kΩ and 1 MΩ.

Electrophysiological data were acquired using an Intan RHD USB interface board and 64‐channel RHD recording headstages with a sampling rate of 20 kHz.

Videos of animal behavior were recorded using a Logitech C922 WebCam at 30 fps. Positions and head directions were tracked with custom software based on YOLOv5.

### Data Preprocessing

Electrophysiological data were processed with a 300 Hz high‐pass filter and spike detection using NDManager.^[^ ^70^ ^]^ The extracted spikes were automatically sorted with Klustakwik,^[^ ^71^ ^]^ followed by further manual sorting using Kluster. Units were automatically classified into two categories: putative pyramidal cells and putative interneurons using CellExplorer,^[^ ^72^ ^]^ based on the peak widths of spike waveforms and autocorrelograms. Only putative pyramidal cells were included in further analyses.

To extract LFP data, electrophysiological data were downsampled to 1250 Hz and notch filtered between 48 and 52 Hz. Channels with noise contamination were excluded.

### Histology

Rats were deeply anesthetized with sodium pentobarbital solution (1%, 0.1 g kg^−1^, i.p.). Electrolytic lesions were made at the recording sites by passing currents (≈20 µA for 5 s on 6 channels with high‐density spike recorded). The rats were then transcardially perfused with 0.9% saline followed by 4% paraformaldehyde (PFA) in 0.1 M phosphate buffer (PB, pH = 7.4). Brains were carefully removed, post‐fixed in 4% PFA for 12 h, and subsequently cryoprotected by sequential immersion in 20% and 30% sucrose in 0.1 M PB. The fixed brains were cut into 80 µm thick frozen slices. Brain slices were stained with Cresyl Violet. Rats with poor positions to tetrode tips and an insufficient number of recorded pyramidal cells were excluded from further analysis.

### Behavioral Analysis

Preferences of a rat in the Free Choice sessions were calculated with the following formula:

(1) $$Preference=\frac{n_{changed}-n_{unchanged}}{n_{changed}+n_{unchanged}}$$

where *n_changed_* is the number of trials in which the rat nose‐poked and received a reward on the changed reward side, and *n_unchanged_* is the number of trials in which the rat nose‐poked and received a reward on the unchanged reward side.

As shown by the distribution of the preferences for the changed reward side in stage 3 (Figure S1B, Supporting Information), there is a clear division in the distribution of preferences. Thus, we set −25% and 25% as the criteria of clear preference and no preference empirically.

We used a widely used state‐space model of learning to estimate the real‐time preferences in the Free Choice sessions of LOS periods for each rat.^[^ ^6^, ^19^ ^]^ This model uses the choices of each trial to estimate the rat's preference for a certain side of the maze, along with confidence bounds on that estimated preference.

### Firing Rate Maps and Template Construction

Data recorded in the T‐maze were used to build both 2D and 1D rate maps of each unit. Data with running speeds less than 5 cm s^−1^ and adjacent to the nose‐pokes were discarded.

2D rate map: To compute firing rate maps of each cell, spikes of each unit and occupancy of rats were sorted into 2 cm × 2 cm bins to generate spike distribution and occupancy distribution. A Gaussian kernel with σ = 4 cm was used to smooth both spike distribution and occupancy distribution. Then we divided the spike distribution of each unit by the occupancy distribution to construct 2D rate maps of each unit.

1D rate map: To construct the 1D rate map according to the shape of our maze, we divided the entire trajectory into the T‐maze into four parts: left & right outbound: from the start of the central arm to one of the horizontal arm's terminals, and left & right inbound: returning to the start region along the side arms. The runs of rats in the central arm were divided into two categories according to the following turns (turning left or turning right). The Gaussian kernel with σ = 4 cm were also applied to the 1D spike distribution and occupancy map. The rate maps were computed by dividing the smoothed spike distribution by the smoothed occupancy map and connected head‐to‐tail: inverted left inbound – inverted left outbound – right outbound – right inbound.

Spatial information: We computed spatial information according to connected 1D rate maps. According to previous research, the following formula was used to compute spatial information:

(2) $$I=\sum_{i=1}^{N}p_{i}\frac{\lambda_{i}}{\lambda }\log_{2}\frac{\lambda_{i}}{\lambda }$$

where *I* is the information rate of the cell in *bits*/*spike*. *i* is the number of spatial bins. *N* is the number of spatial bins. *p_i_* is the probability density for the rat being at the *i*th bin. λ_*i*_ is the mean firing rate when the rat is at the *i*th position bin. $\lambda =\sum_{i=1}^{N}\lambda_{i}p_{i}$ is the overall mean firing rate of the cell. Units with spatial information less than 0.25 bits/spike were discarded from further analysis.

Then we built a 1D rate map template for each part of the trajectory. Cells active on the corresponding part of the trajectory (having a peak firing rate exceeding 3 standard deviations (SD) above their mean firing rate) were included in the template. For cells with two peaks, the higher peak was considered. Cells with 3 or more peaks were excluded. The four templates were used for Bayesian decoding.

### Remapping Analysis

To analyze remapping of firing rate maps of each unit, we calculated 1D firing rate maps using data respectively from the Forced Entry session and the Free Choice session. Then we computed Pearson correlation coefficients between the Forced Entry rate map and the corresponding Free Choice rate map. Units with coefficients less than 0.5 were discarded.

### LFP Analysis and Ripple Detection

The PSD of single‐channel LFPs was calculated using the multi‐taper estimation in the Chronux toolbox.^[^ ^73^ ^]^ For ripple detection, the channel with the highest mean power in the ripple frequency band (100–250 Hz) was selected. The Hilbert amplitude was then calculated. Putative ripples were identified at time points exceeding 2 SD, and those without peaks exceeding 3 SD were discarded. Ripples with intervals shorter than 30 ms were merged, while those with a duration shorter than 20 ms or longer than 400 ms were excluded. Ripples with a duration exceeding 100 ms were defined as long ripples. During the online sessions, ripple detection was restricted to periods when nose pokes were triggered, i.e., when the animal was consuming the sucrose water. During these periods, the animal was required to remain stationary. Ripple rates were calculated by dividing the number of ripples detected during these sucrose water consumption periods by the total duration of these periods. In both Rest sessions, the animal's movement was restricted by being placed on a flower pot tray, preventing locomotion. As a result, the animal was considered to be continuously immobile throughout the session. Therefore, ripple rates for Rest sessions were calculated by dividing the total number of ripples by the full duration of the session. For the Sleep session, only the NREM epochs were used to calculate ripple rate.

### Sleep Analysis

In this study, we used custom code modified from an open‐source MATLAB library.^[^ ^74^ ^]^ As in previous studies,^[^ ^65^, ^75^, ^76^, ^77^, ^78^ ^]^ we combined LFP data from the dCA1 region and head movement data to determine the stages of wakefulness and sleep. We divided the Sleep session into 5‐s epochs and calculated the relative power of the LFP in the theta and delta frequency bands, as well as the sum of variances across the three‐axis accelerometer data for each epoch. By setting a threshold for the sum of variances of the three‐axis accelerometer data, we classified time epochs into immobile and moving epochs (Figure S5B, Supporting Information). Sleep stages (NREM or rapid eye movement (REM) sleep) were determined based on the relative power of delta and theta bands of continuous immobility states lasting at least 20 s (Figure S5A,D, Supporting Information). By setting the threshold of relative delta and theta band power, epochs were classified as delta‐dominant or theta‐dominant. Sleep epochs that were theta‐dominant but not delta‐dominant were classified as REM sleep, while other sleep epochs were classified as NREM sleep. The sleep analysis method used in this study showed a high level of concordance with another method widely used in previous studies (Figure S10, Supporting Information).^[^ ^60^, ^79^, ^80^, ^81^, ^82^ ^]^ The theta/delta power ratio during short immobility and sleep states further supports the validity of the sleep analysis method (Figure S5C, Supporting Information). Total sleep duration and the proportions of each sleep stage were then calculated based on the classified states of each epoch.

### PBEs

According to previous studies, SWRs and replays in the dorsal hippocampus tend to occur during reward consumption, awake rest, and NREM sleep, so we analyzed PBEs and replays during these stages.

PBEs were detected as described in previous studies.^[^ ^25^ ^]^ Spikes of all recorded units were combined into MUAs. The MUAs were binned into 10‐ms bins and were smoothed by a Gaussian kernel with σ of two bins and normalized from 0 to 1. A putative PBE was defined as a period with a peak of normalized MUA spike counts exceeding a threshold of 0.35. Its start and end times were defined as time points crossing the threshold of 0.15. Adjacent putative PBEs with gaps < 30 ms were combined. Putative PBEs with a duration of less than 80 ms or greater than 500 ms were discarded.

To calculate PBE rates during the online sessions, we detected PBEs exclusively within the periods when nose pokes were triggered, that is, during sucrose consumption, when the animal was stationary. PBE rates were then computed by dividing the number of PBEs detected within these periods by the total duration of these periods. In both Rest sessions, the animal's movement was restricted by being placed on a flower pot, preventing locomotion. As a result, the animal was considered to be continuously immobile throughout the session. Therefore, ripple rates for Rest sessions were calculated by dividing the total number of ripples by the full duration of the session. For sleep sessions, PBE detection was restricted to NREM sleep epochs. The PBE rate was calculated by dividing the number of PBEs during NREM sleep by the total duration of NREM sleep.

### Spatial Representation Map and Activation Preference

To visualize the distribution of spatial representation expressed by the spikes in PBEs during certain time epochs, we superimposed 2D rate maps of activated place cells, with each map weighted by the number of spikes fired within the time range. Then we normalized the resulting rate map to values between 0 and 1.

After that, we computed the activation preference of left and right trajectories. Left run cells were defined as cells participating in left outbound and inbound templates, so as right run cells. The spikes fired by left run cells and right run cells were counted in all PBEs within a certain time range. The activation rate of each side was the fraction of spikes of corresponding cells over all spikes fired by all template cells, normalized (divided) by the fraction of the number of corresponding cells over the number of all template cells. Then, the activation preference of cells corresponding to the changed reward side was calculated by the following equation:

(3) $$Activation\;preference=\frac{rate_{changed}-rate_{unchanged}}{rate_{changed}+rate_{unchanged}}$$

### Pairwise Coactivation Analysis

We analyzed the coactivation levels of template cell pairs during the Free Choice session, two rest sessions, and the Sleep session.

In the Free Choice session, we focused on the reward consumption in the Start Region between two trials and allocated reward consumption periods to the following trials (forward allocation) or to previous trials (backward allocation).

For a template cell pair (of either the changed reward side or the unchanged reward side), we calculated the coactivation probabilities of trials corresponding to ipsilateral (I) choices and contralateral (C) choices, respectively (based on forward or backward allocation). The coactivation probabilities of a template cell pair in I and C trials were calculated as the number of PBEs during which both cells were active, divided by the total number of PBEs in that trial type. Similarly, the activation probability of a single template cell in I or C trials was defined as the number of PBEs in which the cell was active, divided by the total number of PBEs in that trial type.

To exclude cells or cell pairs that rarely fired during PBEs, we included in our analysis only those cell pairs whose coactivation probability was at least 0.01 in either I or C trials, or whose individual activation probabilities were both at least 0.01.

To determine whether activation of template cell pairs was stronger in I trials compared to C trials, we computed and averaged the z‐score of the difference between coactivation probabilities during PBEs in I and C trials for cell pairs of two templates. For each template cell pair, the coactivation probability of each trial type is computed with the following formulas:

(4) $$\hat{p_{I}}=\frac{n_{I}}{N_{I}}$$

(5) $$\hat{p_{C}}=\frac{n_{C}}{N_{C}}$$

where *n_I_* and *n_C_* are the numbers of PBEs in I trials and C trials, in which both template cells were active. *N_I_* and *N_C_* are the total numbers of PBEs in I trials and C trials. To compare the difference in these probabilities across different behavioral periods, we normalized $\hat{p_{diff}}=\hat{p_{I}}-\hat{p_{C}}$ to a Z score according to the estimated standard error of the difference based on a binomial distribution:

(6) $$\hat{p}=\frac{n_{I}+n_{C}}{N_{I}+N_{C}}$$

(7) $$\mathrm{stde}\mathrm{rr}=\sqrt{\hat{p}\left(1-\hat{p}\right)\left(\frac{1}{N_{I}}+\frac{1}{N_{C}}\right)}$$

Then the Z score of each cell pair is $\hat{p_{diff}}/stderr$, and we calculated the average of Z scores of all cell pairs for each recording day.

For the sleep session and awake rest sessions, we analyze the pairwise coactivation of template cells corresponding to the changed reward side and the unchanged reward side of the maze. Similar to the pairwise coactivation analysis in the online session, we calculated the activation probability of each template cell and the coactivation probability of each template cell pair by dividing the number of PBEs of activation or coactivation by the total number of PBEs. The inclusion criteria of template cell pairs are the same as the analysis of the online state. Then we normalize the number of PBEs in which the template cell pair (cell *i* and cell *j*) coactivated, *K_ij_*, to a Z score using expectation and variance based on a hypergeometric distribution:

(8) $$\hat{K_{ij}}=\frac{n_{i}n_{j}}{N}$$

(9) $$Var\left(K_{ij}\right)=\frac{n_{i}n_{j}}{N}\left(1-\frac{n_{j}}{N}\right)\cdot \frac{N-n_{i}}{N-1}$$

where *n_i_* and *n_j_* are the numbers of PBEs in which template cell pair *i* and *j* activated, and *N* is the total number of PBEs. The Z score of the coactivation times of each template cell pair is $\left(K-\hat{K}\right)/\sqrt{Var(K)}$ for each template cell pair. We then computed the difference between the average Z score of template cell pairs of the changed reward side template and that of the unchanged reward side template to indicate the bias of cell pair coactivation between the two templates.

### Decoding Replays

Replays were identified by a Bayesian decoding method as described in previous studies.^[^ ^21^ ^]^ Firing rate maps in each template were used as the firing probabilities for the cell at each location of the trajectory. PBEs with at least four active template cells were defined as candidate replay events for the corresponding region. For each candidate event, we performed Bayesian decoding on each 20‐ms time bin with 10‐ms steps according to the following formula:

(10) $$P\left(x_{p}|n_{t}\right)=K_{t}\left\{\prod_{i=1}^{N}\lambda_{i}{\left[x_{p}\right]}^{n_{i,t}}\right\}e^{-\tau \sum_{i=1}^{N}\lambda_{i}\left[x_{p}\right]}$$

where τ is the duration of the time bin (20 ms), λ_i_[x_p_] is the firing rate of the i‐th neuron at x_p_ on the track corresponding to the template, K_t_ is a normalization constant to ensure that the sum of probabilities across all position bins equals 1, n_t_ is the number of spikes fired by each neuron in that time bin. The decoded position of each time bin is the spatial bin with the maximum posterior probability.

To estimate the significance of putative replays, we performed linear regression between decoded positions and time bins. We shuffled the decoded spatial locations corresponding to the time bins and performed linear regression 1000 times, and defined the proportion of shuffled *R* values larger than the actual *R* values as the *p* value. A candidate event was considered a replay if *p* < 0.05 and *R* > 0.6. Replays with positive *R* values were defined as forward replays, and replays with negative *R* values were defined as reverse replays.

We applied Bayesian decoding and linear regression to each putative replay event using all four templates. If the decoding result from any template met the criteria of *p* < 0.05 and *R* > 0.6, the event was classified as a replay of the region corresponding to that template. In cases where multiple templates satisfied these criteria, the template with the highest *R* value was selected, and the putative replay was assigned to the corresponding region. The PBE rate and replay rate were defined as the number of PBEs and replays divided by their corresponding time durations, respectively.

### Replay Preference and Replay Direction

For each stage, to examine whether replays were biased to the region with changed reward, we calculated the preference of replays for both the outbound and inbound templates of the corresponding side:

(11) $$\mathrm{Preference}=\frac{n_{s1}-n_{s2}}{n_{s1}+n_{s2}}$$

where n_s1_ and n_s2_ are the numbers of replays corresponding to the target side and the other side, respectively. Depending on the template region and whether the replays are forward or reverse, we categorize replays into pointing toward rewards and away from rewards. For left and right outbound templates, forward replays pointed toward rewards while reverse replays pointed away from rewards. For left and right inbound templates, forward replays pointed away from rewards, while reverse replays pointed toward rewards. For outbound and inbound templates corresponding to the changed reward, we calculated percentages of replays pointing toward the changed reward and those pointing away from the changed reward. To illustrate replay directions, we constructed replay vectors that point from the decoded position of the first time bin to the decoded position of the last time bin.

### Trial‐by‐Trial Choice Prediction

We investigated whether replays in the Start region of a Free Choice session could predict the animal's subsequent or previous choices. We calculated replay bias during each reward consumption in the Start region. Then we predicted that the animal would choose the left (or right) side of the maze on the next trial (or previous trial) if the replays were biased toward the left (or right) side. Trials with no significant replay or with equal numbers of replays corresponding to the left and right side were excluded. The accuracy of prediction was defined as the percentage of trials predicted correctly. To assess whether the prediction accuracy significantly exceeded the chance level of 50%, we conducted one‐tailed binomial tests on the trial‐by‐trial prediction results for each period separately, under the null hypothesis that the prediction accuracy was 50%.

### Statistical Analysis

All statistical analyses and visualizations were performed using MATLAB 2023b, Python 3.8, and GraphPad Prism 8. For line plots and histograms with replication, data were presented as means ± SEM. In box plots with whiskers, the center line indicated the median, the box represented the 25th–75th percentiles, and the whiskers denoted the minimum and maximum values. In box plots without whiskers, the floating bar represented the minimum and maximum values, and the center line indicated the median. In violin plots, the solid lines indicate the median, and the dashed lines represent the 25th and 75th percentiles. For preference curves, the solid line indicated the estimated preference value, and the shaded area represented the 5%–95% confidence interval.

Comparisons between one group and a specific value were conducted using a two‐sided one sample t‐test. Comparisons between two groups were conducted using a two‐sided two sample t‐test. Comparisons among three or more groups were conducted using one‐way ANOVA with Tukey post hoc analysis. Comparisons of accuracy with chance level were conducted using a one‐sided binomial test. Significance thresholds were set as follows: *p* < 0.05 (*), *p *< 0.01 (**), and *p* < 0.001 (***). Sample sizes (n), p‐values, and F‐values (of one‐way ANOVA) were reported in the figure legends.

## Conflict of Interest

The authors declare no conflict of interest.

## Author Contributions

J.T., Y.W., and M.Y. conceptualized and designed the study; J.T. and Y.X. designed the custom drive and electrode interface board (EIB); J.T. and Y.X. analyzed the electrophysiological and behavioral data; J.T. and Y.Z. performed in vivo electrophysiological experiments; L.W. assisted with the processing of behavioral videos; S.S. helped improve the design of electrodes; S.S., L.M., S.L., and N.L. assisted with surgeries and electrophysiological experiments under the supervision of Y.W. and M.Y.; Y.X. and J.W. assisted with data preprocessing; N.L., X.Q., T.W., K.C., and S.C. assisted with behavioral and histological experiments under the supervision of Y.W. and M.Y.; J.T. wrote the original manuscript; Y.W. and M.Y. revised the manuscript with contributions from all authors. All authors read and approved the manuscript.

## Supporting information



Supporting Information

## Data Availability

The data that support the findings of this study are available from the corresponding author upon reasonable request.
