# Supplementary material for: Long‐Term Memory Updating Parallels Altered Awake and Sleep Hippocampal Replays
Source: Adv Sci (Weinh). 2025 Nov 5;13(3):e16480. doi: 10.1002/advs.202416480 (PMC12806511; doi:10.1002/advs.202416480)
Supplement: Supplementary file 1 — Supporting Information [file ADVS-13-e16480-s001.docx]

Supporting Information

**Long-term memory updating parallels altered awake and sleep hippocampal replays**

*Jifu Tong, Yuanwei Xing, Yawen Zheng, Linshu Wang, Shan Shao, Jiao Wu, Longyu Ma, Shuting Liu, Naizheng Liu, Xuetao Qi, Ting Wang, Kun Cui, Shuang Cui, You Wan^*^, Ming Yi^*^*

**Table S1. Number of place cells.** 1816 place cells were recorded in the CON, LOS, and REV periods (6 rats), and 406 place cells were recorded in the NU period (4 out of 6 rats). Rat 5 and Rat 8 were excluded from the analysis of electrophysiological data because of the poor positions of tetrode tips and insufficient amounts of place cells. The number of place cells listed in the table refers to the place cells simultaneously recorded during each corresponding period, with no double-counting across sessions.

| Number of place cells | Rat 1 | Rat 2 | Rat 3 | Rat 4 | Rat 6 | Rat 7 |
| --- | --- | --- | --- | --- | --- | --- |
| CON period | 99 | 100 | 106 | 103 | 105 | 120 |
| NU period | - | 97 | 92 | - | 101 | 116 |
| LOS period | 95 | 94 | 99 | 95 | 102 | 119 |
| REV period | 93 | 93 | 92 | 95 | 100 | 106 |


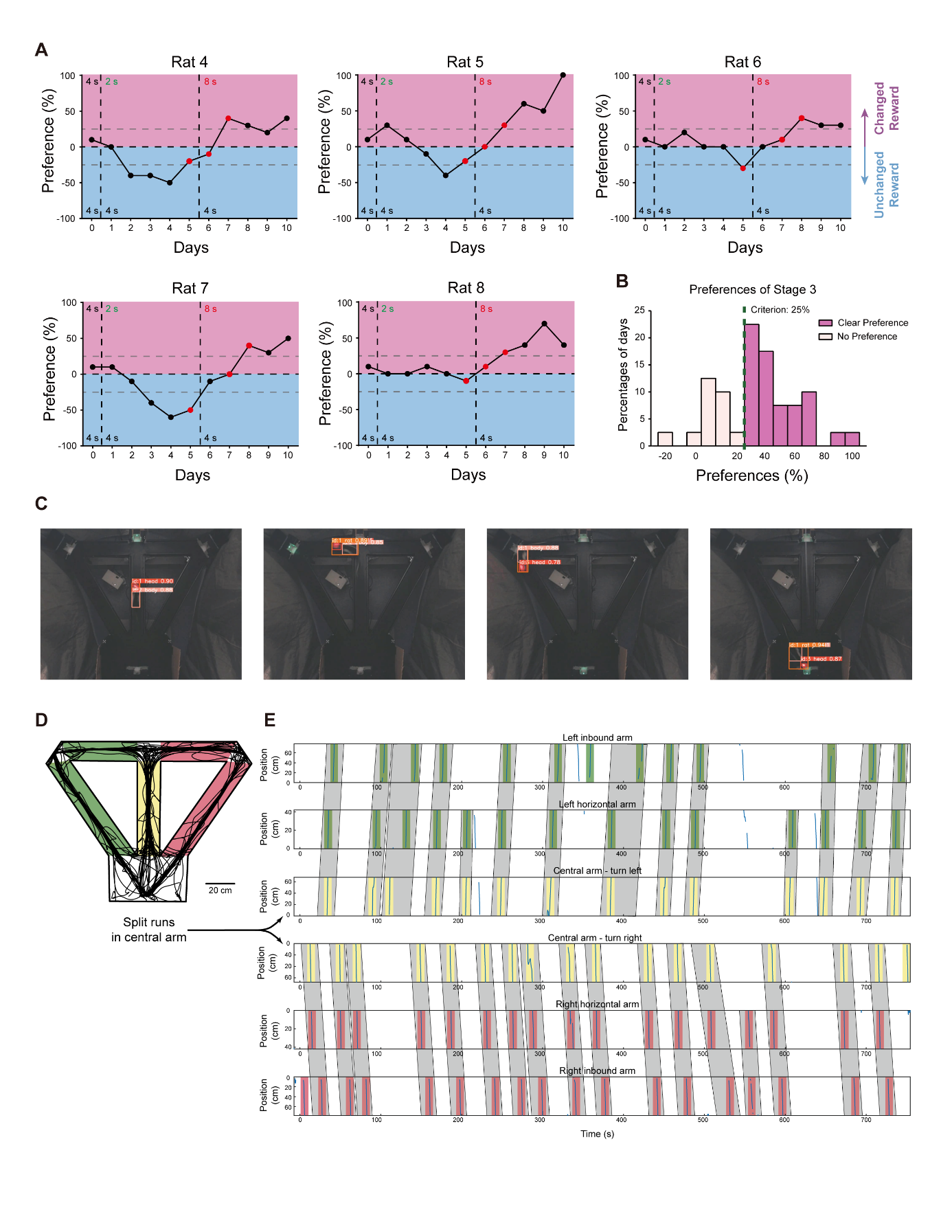


Figure S1. Behavioral performance of rats. (A) Behavioral performances of the other 5 rats. Purple region: preference to the changed reward side; blue region: preference to the unchanged reward side. Horizontal dashed lines indicate the boundary of clear preferences (±25%). Vertical dashed lines indicate the boundary between stage 1, stage 2, and stage 3. Red dots indicate CON, LOS, and REV periods. (B) Distribution of preferences for all days in stage 3. The dashed line indicates 25%, which is the boundary between clear preferences and not significant preferences. (C) Representative frames of our custom animal tracking algorithm. (D) Representative tracks of a rat’s head in a Free Choice session. (E) Top to bottom: representative 1D running tracks in the left inbound arm, left horizontal arm, central arm (followed by turning left), central arm (followed by turning right), right horizontal arm, and right inbound arm. Shaded region: continuous runs of single trials in the Free Choice sessions.


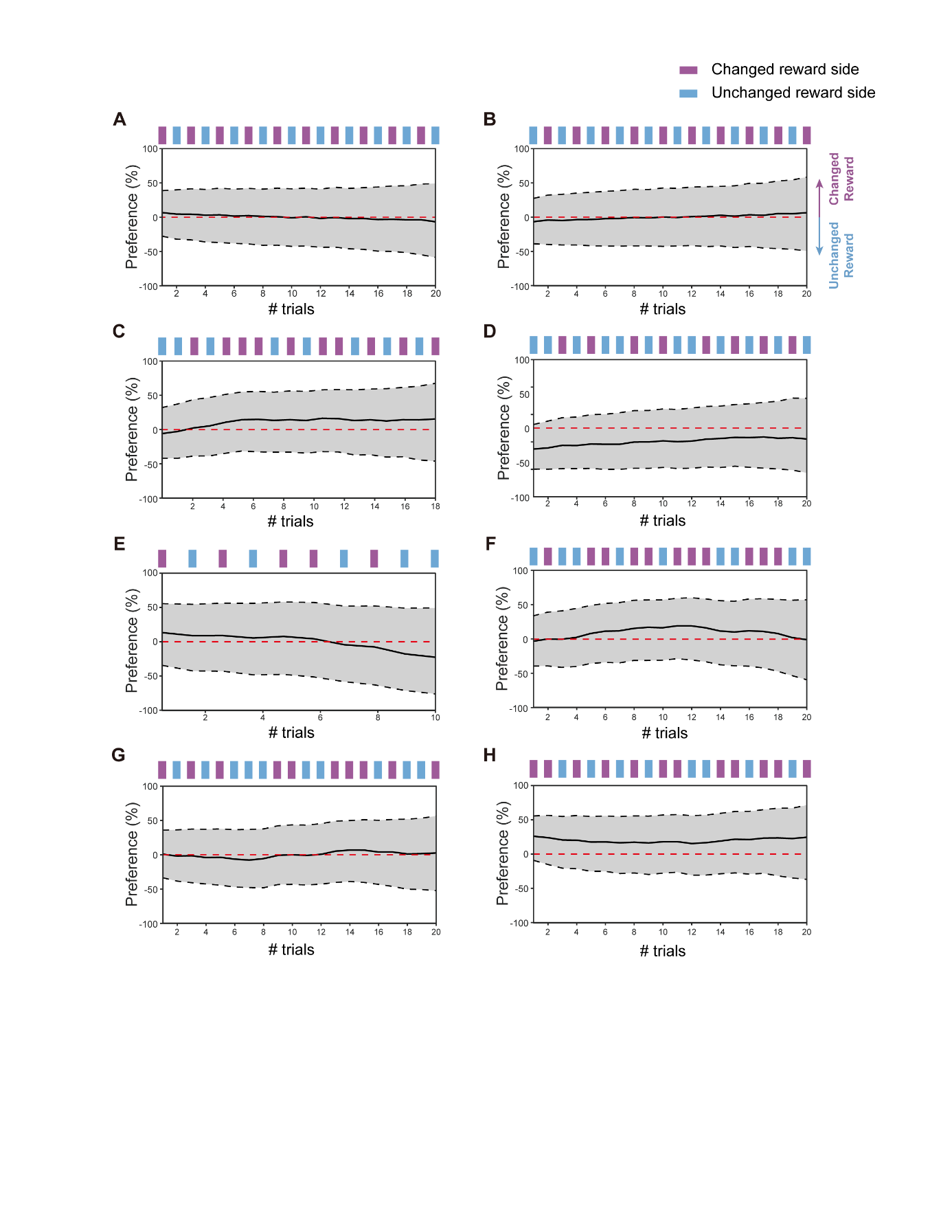


Figure S2. Preferences within a single day stay steady. (A-H) Real-time preferences to the changed reward side in the Free Choice session of LOS periods of each rat. The squares above the coordinate axes represent the choices of rats. The solid line indicates the estimated preference value, and the shaded area represents the 5%–95% confidence interval.


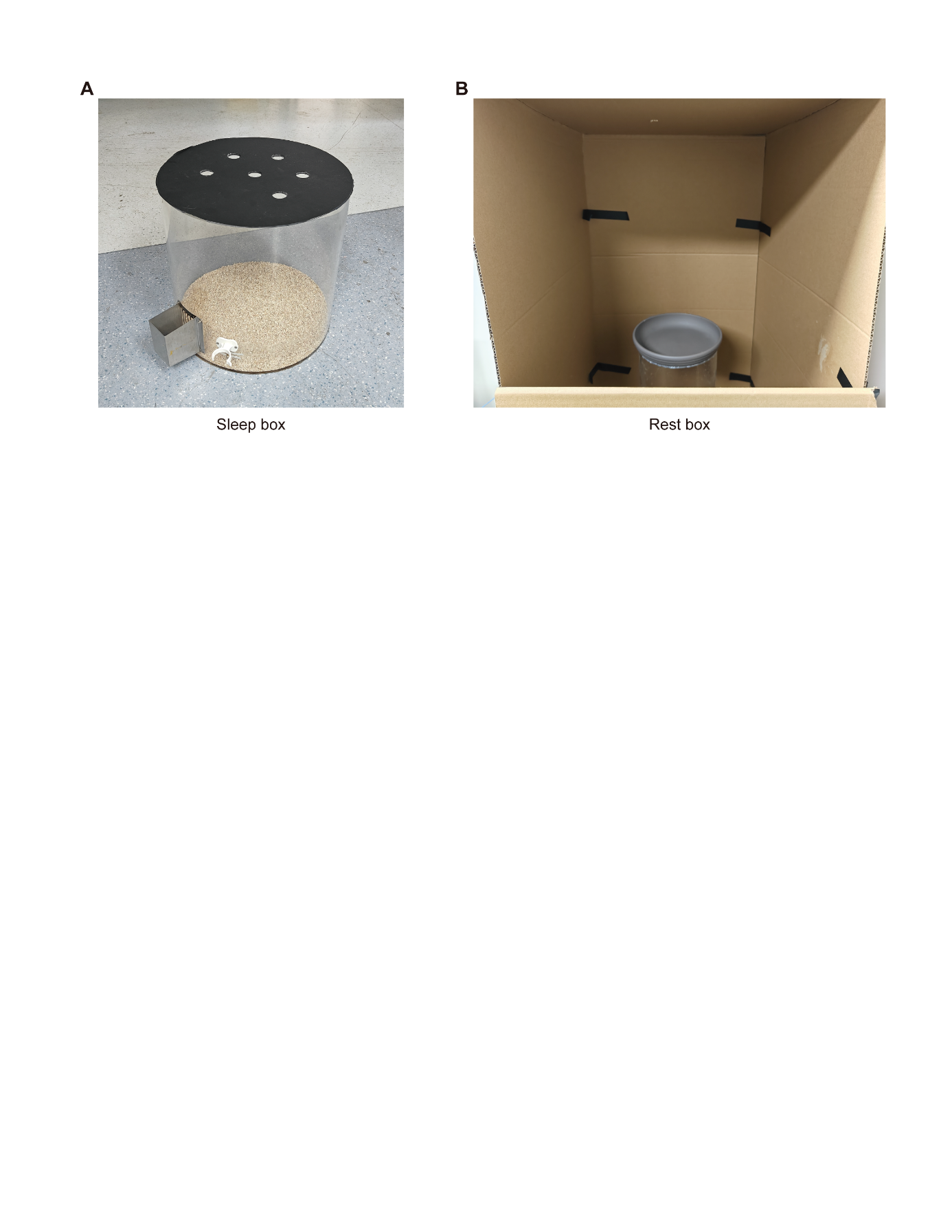


Figure S3. Apparatus used in the behavioral paradigms. (A) Sleep box is an acrylic cylinder with a diameter of 40 cm and height of 40 cm. (B) Rest box is an elevated flowerpot tray located within an enclosed box.


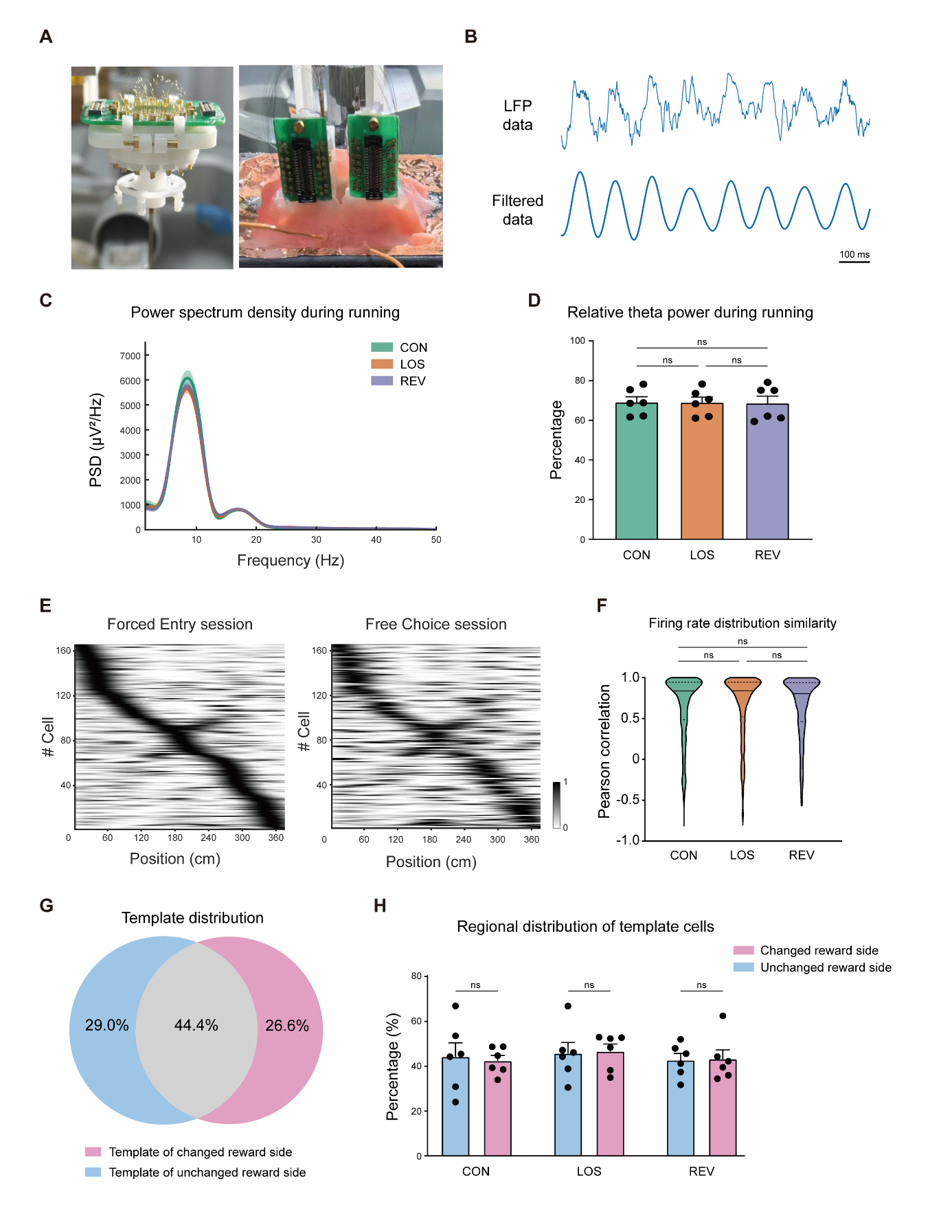


Figure S4. Theta-band powers during exploration stay constant. (A) Unilateral (left) and bilateral tetrode drives implanted into the hippocampus. (B) Representative theta oscillation in the CA1 region. Upper, wide-band LFP data during continuous running in the maze; lower, the same data filtered with theta band (4 – 13 Hz). Scale bar, 100 ms. (C) Representative average (mean ± SEM) PSD data of LFP segments during running in the maze in the three periods of memory updating. (D) Relative mean theta-band power during running segments in the maze in CON, LOS, and REV periods. One-way ANOVA with Tukey post hoc analysis: F (2, 15) = 0.006159, p = 0.9939; CON vs. LOS: p = 0.9991; CON vs. REV: p = 0.9934; LOS vs. REV: p = 0.9974. Data are presented as mean ± SEM (n = 6 rats). (E) Left: head-to-tail connected 1-dimensional firing rate maps of place cells computed by the data from the Forced Entry session, sorted by the place field positions. Right: head-to-tail connected 1-dimensional firing rate maps of place cells computed by the data from the Free Choice session, in the same order as the left one. (F) Pearson correlations between firing rate maps of place cells computed by the data from the Forced Entry sessions and Free Choice sessions in the CON, LOS, and REV periods. One-way ANOVA with Tukey post hoc analysis: F (2, 1813) = 0.3403, p = 0.7116; CON vs. LOS: p = 0.9790; CON vs. REV: p = 0.8164; LOS vs. REV: p = 0.7080. The solid line indicates the median, and the dashed lines represent the 25th and 75th percentiles (n_CON_ = 633 cells, n_LOS_ = 604 cells, n_REV_ = 579 cells). (G) Proportions of template cells only in the changed reward side template, only in the unchanged reward side template, and in both templates among all template cells. (H) Proportions of template cells in the changed reward side template and unchanged reward side template in all place cells across the CON, LOS, and REV periods. Two sample t-test for CON, LOS, and REV periods, respectively: CON period, p = 0.7905; LOS period, p = 0.8766; REV period, p = 0.9219. Data are presented as mean ± SEM (n = 6 rats). ***, p < 0.001; **, p < 0.01; *, p < 0.05; ns, p > 0.05.


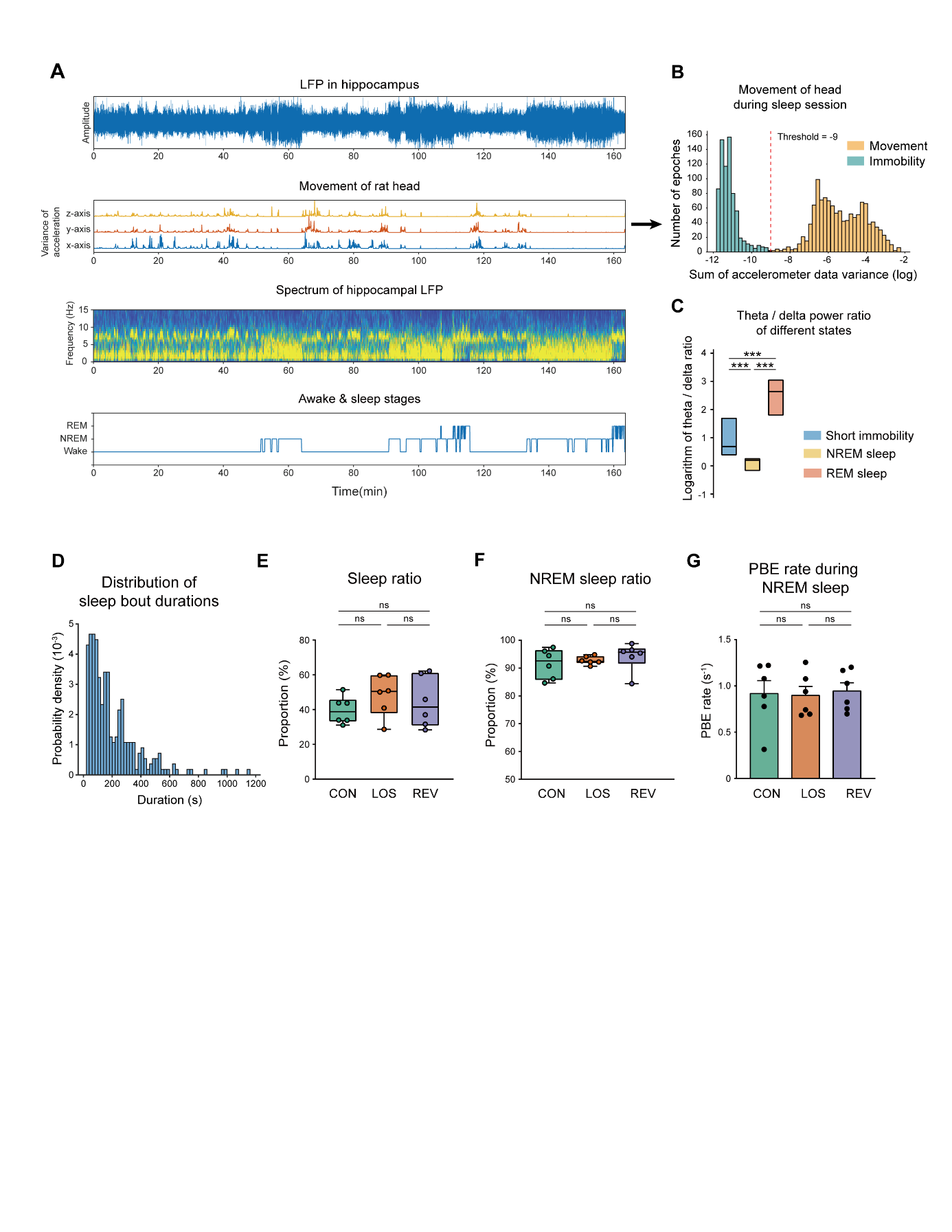


Figure S5. Sleep architecture remained stable. (A) Sleep stages of a representative sleep recording. From upper to lower, first: normalized LFP data in the CA1 region; second: variances of three-axis accelerometer data indicating head movement; third: time-frequency spectrogram of LFP data in the CA1 region; fourth: stages of sleep epochs. (B) Distribution of sums of variances of three-axis accelerometer data indicating head movement of the rats in the Sleep sessions. The dashed line indicates the boundary of head movement and immobility. (C) Logarithm of the theta/delta power ratio during short immobility (<20 s, excluded according to the sleep analysis method), NREM sleep, and REM sleep. One-way ANOVA with Tukey post hoc analysis: F (2, 51) = 207.7, p < 10^-4^; CON vs. LOS: p < 10^-4^; CON vs. REV: p < 10^-4^; LOS vs. REV: p < 10^-4^. Floating bars represent the minimum and maximum values, and the center line indicates the median (n = 18 days). (D) Distribution of sleep bout durations derived from the sleep analysis method. (E) Proportions of sleep in the first two hours of Sleep sessions. One-way ANOVA with Tukey post hoc analysis: F (2, 15) = 0.8132, p = 0.4621; CON vs. LOS: p = 0.4304; CON vs. REV: p = 0.7777; LOS vs. REV: p = 0.8260. The center line indicates the median, the box represents the 25th–75th percentiles, and the whiskers show the minimum and maximum values (n = 6 rats). (F) Proportions of NREM sleep within the first 30 minutes of sleep during the CON, LOS, and REV periods. One-way ANOVA with Tukey post hoc analysis: F (2, 15) = 0.5446, p = 0.5911; CON vs. LOS: p = 0.8837; CON vs. REV: p = 0.5627; LOS vs. REV: p = 0.8395. The center line indicates the median, the box represents the 25th–75th percentiles, and the whiskers show the minimum and maximum values (n = 6 rats). (G) PBE rates during NREM sleep in CON, LOS, and REV periods. One-way ANOVA with Tukey post hoc analysis: F (2, 15) = 0.04731, p = 0.9539; CON vs. LOS: p = 0.9924; CON vs. REV: p = 0.9809; LOS vs. REV: p = 0.9502. Data are presented as mean ± SEM (n = 6 rats). ***, p < 0.001; **, p < 0.01; *, p < 0.05; ns, p > 0.05.


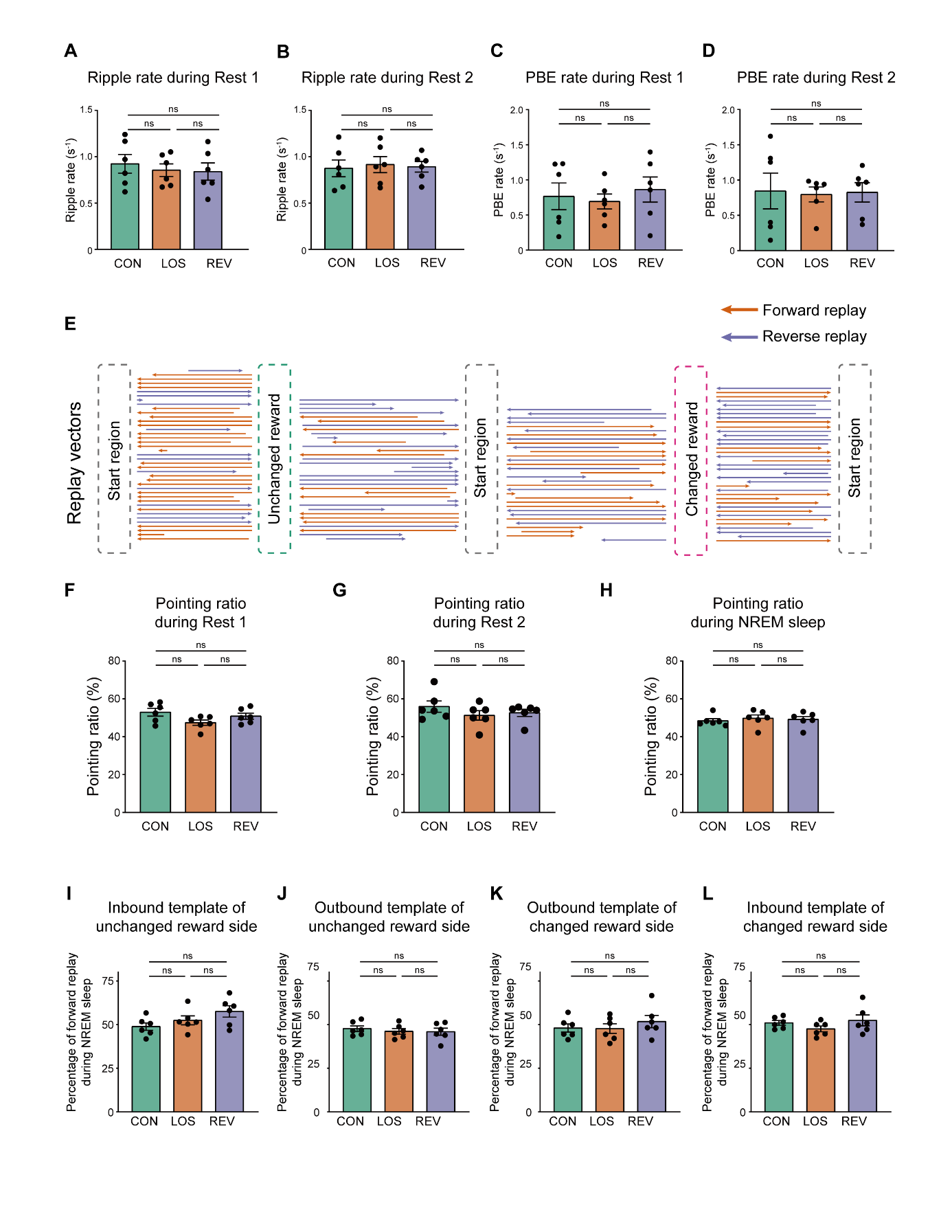


**Figure S6. Pointing ratios of replays during the offline states stage were unchanged.** **(A-B)** Ripple rates during Rest 1 session (A) and Rest 2 session (B) in the CON, LOS, and REV periods. One-way ANOVA with Tukey post hoc analysis, Rest 1 session: F (2, 15) = 0.2483, p = 0.7833; CON vs. LOS: p = 0.8517; CON vs. REV: p = 0.7893; LOS vs. REV: p = 0.9924; Rest 2 session: F (2, 15) = 0.06451, p = 0.9378; CON vs. LOS: p = 0.9322; CON vs. REV: p = 0.9876; LOS vs. REV: p = 0.9767. Data are presented as mean ± SEM (n = 6 rats). **(C-D)** PBE rates during Rest 1 session (C) and Rest 2 session (D) in the CON, LOS, and REV periods. One-way ANOVA with Tukey post hoc analysis, Rest 1 session: F (2, 15) = 0.2734, p = 0.7645; CON vs. LOS: p = 0.9466; CON vs. REV: p = 0.9075; LOS vs. REV: p = 0.7458; Rest 2 session: F (2, 15) = 0.01936, p = 0.9808; CON vs. LOS: p = 0.9792; CON vs. REV: p = 0.9968; LOS vs. REV: p = 0.9922. Data are presented as mean ± SEM (n = 6 rats). **(E)** Representative distribution of replay vectors within a sleep bout. From left to right, the regions correspond to the left inbound, left outbound, right outbound, and right inbound segments of the rat's trajectory. Orange arrows indicate forward replays; violet arrows indicate reverse replays. Replay vectors pointing toward the reward are represented as arrows directed toward the changed reward region. **(F-H)** Ratios of replays pointing to the changed reward site during Rest 1 session (F), Rest 2 session (G), and Sleep session (H) in the CON, LOS, and REV periods. One-way ANOVA with Tukey post hoc analysis, Rest 1 session: F (2, 15) = 2.655, p = 0.1030; CON vs. LOS: p = 0.0896; CON vs. REV: p = 0.6715; LOS vs. REV: p = 0.3562; Rest 2 session: F (2, 15) = 0.9441, p = 0.4110; CON vs. LOS: p = 0.3993; CON vs. REV: p = 0.6156; LOS vs. REV: p = 0.9243; Sleep session: F (2, 15) = 0.2069, p = 0.8154; CON vs. LOS: p = 0.7992; CON vs. REV: p = 0.9363; LOS vs. REV: p = 0.9529. Data are presented as mean ± SEM (n = 6 rats). **(I)** Percentage of forward replays corresponding to the inbound template of the unchanged reward side during NREM sleep. One-way ANOVA with Tukey post hoc analysis: F (2, 15) = 2.636, p = 0.1045; CON vs. LOS: p = 0.6227; CON vs. REV: p = 0.0892; LOS vs. REV: p = 0.3950. **(J)** Percentages of forward replays corresponding to the outbound template of the unchanged reward side during NREM sleep. One-way ANOVA with Tukey post hoc analysis: F (2, 15) = 0.2899, p = 0.7525; CON vs. LOS: p = 0.8144; CON vs. REV: p = 0.7692; LOS vs. REV: p = 0.9963. **(K)** Percentages of forward replays corresponding to the outbound template of the changed reward side during NREM sleep. One-way ANOVA with Tukey post hoc analysis: F (2, 15) = 0.5959, p = 0.5636; CON vs. LOS: p = 0.9966; CON vs. REV: p = 0.6465; LOS vs. REV: p = 0.5986. **(L)** Percentages of forward replays corresponding to the inbound template of the changed reward side during NREM sleep. One-way ANOVA with Tukey post hoc analysis: F (2, 15) = 1.339, p = 0.2916; CON vs. LOS: p = 0.5131; CON vs. REV: p = 0.8886; LOS vs. REV: p = 0.2795. Data are presented as mean ± SEM (n = 6 rats) (I-L). ***, p < 0.001; **, p < 0.01; *, p < 0.05; ns, p > 0.05.


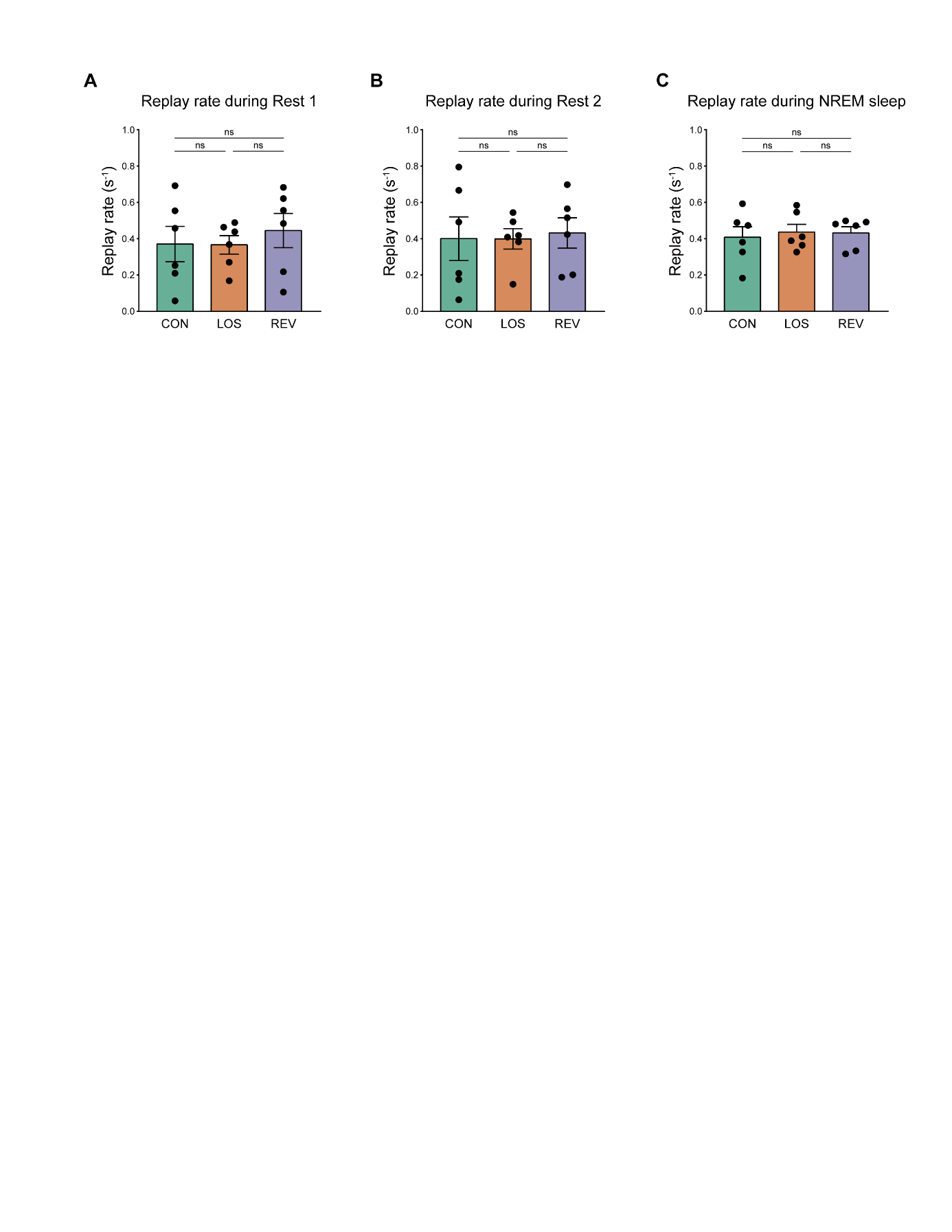


**Figure S7. Replay rates during offline sessions stayed unchanged. (A-C)** Replay rates during Rest 1 session (A), Rest 2 session (B), and Sleep session (C) in CON, LOS, and REV periods. One-way ANOVA with Tukey post hoc analysis, Rest 1 session: F (2, 15) = 0.2793, p = 0.7602; CON vs. LOS: p = 0.9993; CON vs. REV: p = 0.8072; LOS vs. REV: p = 0.7870; Rest 2 session: F (2, 15) = 0.04203, p = 0.9590; CON vs. LOS: p > 0.9999; CON vs. REV: p = 0.9670; LOS vs. REV: p = 0.9648; Sleep session: F (2, 15) = 0.1135, p = 0.8935; CON vs. LOS: p = 0.8974; CON vs. REV: p = 0.9275; LOS vs. REV: p = 0.9970. Data are presented as mean ± SEM (n = 6 rats). ***, p < 0.001; **, p < 0.01; *, p < 0.05; ns, p > 0.05.


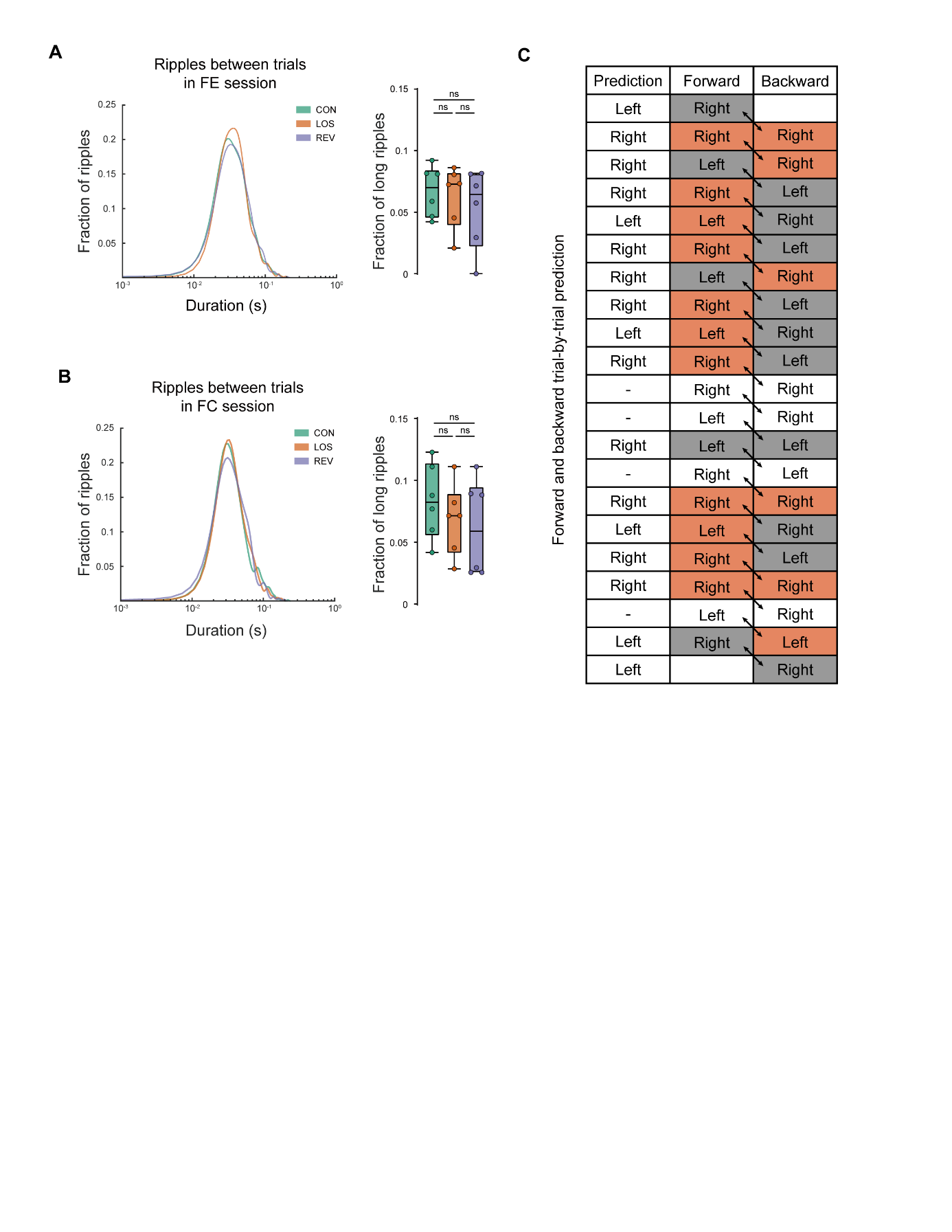


Figure S8. Ripple durations between consecutive trials in online sessions stayed unchanged. (A) Distribution of the durations of ripples (left) and proportions of long ripples (durations > 100 ms) during reward consumption (right) in the start region between consecutive trials in the Forced Entry sessions. Left: one-way ANOVA with Tukey post hoc analysis for the distribution of the ripple durations: F (2, 1049) = 0.2362, p = 0.7897, CON vs. LOS: p = 0.9811, CON vs. REV: p = 0.8780, LOS vs. REV: p = 0.7841 (n_CON_ = 361, n_LOS_ = 344, n_REV_ = 347). Right: one-way ANOVA with Tukey post hoc analysis for the proportions of long ripples: F (2, 15) = 0.4133, p = 0.6688, CON vs. LOS: p = 0.9644, CON vs. REV: p = 0.6584, LOS vs. REV: p = 0.8077, the center line indicates the median, the box represents the 25th–75th percentiles, and the whiskers show the minimum and maximum values (n = 6 rats). (B) Distribution of the durations of ripples (left) and proportions of long ripples during reward consumption (right) in the start region between consecutive trials in the Free Choice sessions. Left: one-way ANOVA with Tukey post hoc analysis for the distribution of the ripple durations: F (2, 956) = 0.5900, p = 0.5545; CON vs. LOS: p = 0.7817, CON vs. REV: p = 0.5436, LOS vs. REV: p = 0.9213 (n_CON_ = 407, n_LOS_ = 296, n_REV_ = 256). Right: one-way ANOVA with Tukey post hoc analysis for the proportions of long ripples: F (2, 15) = 0.6873, p = 0.5181, CON vs. LOS: p = 0.7146, CON vs. REV: p = 0.5023, LOS vs. REV: p = 0.9331, the center line indicates the median, the box represents the 25th–75th percentiles, and the whiskers show the minimum and maximum values (n = 6 rats). (C) Representative predicted and true choices of a rat during the Free Choice session in the LOS period. Three columns from left to right: predicted choices according to regional preferences, true choices following reward consumption in the Start region for forward prediction, and true choices before reward consumption in the Start region for backward prediction. In the right two columns of the table, orange cells indicate consistency with the predicted choice shown in the left column, gray cells indicate inconsistency, and white cells represent trials in which the region preference of the replays was zero. ***, p < 0.001; **, p < 0.01; *, p < 0.05; ns, p > 0.05.


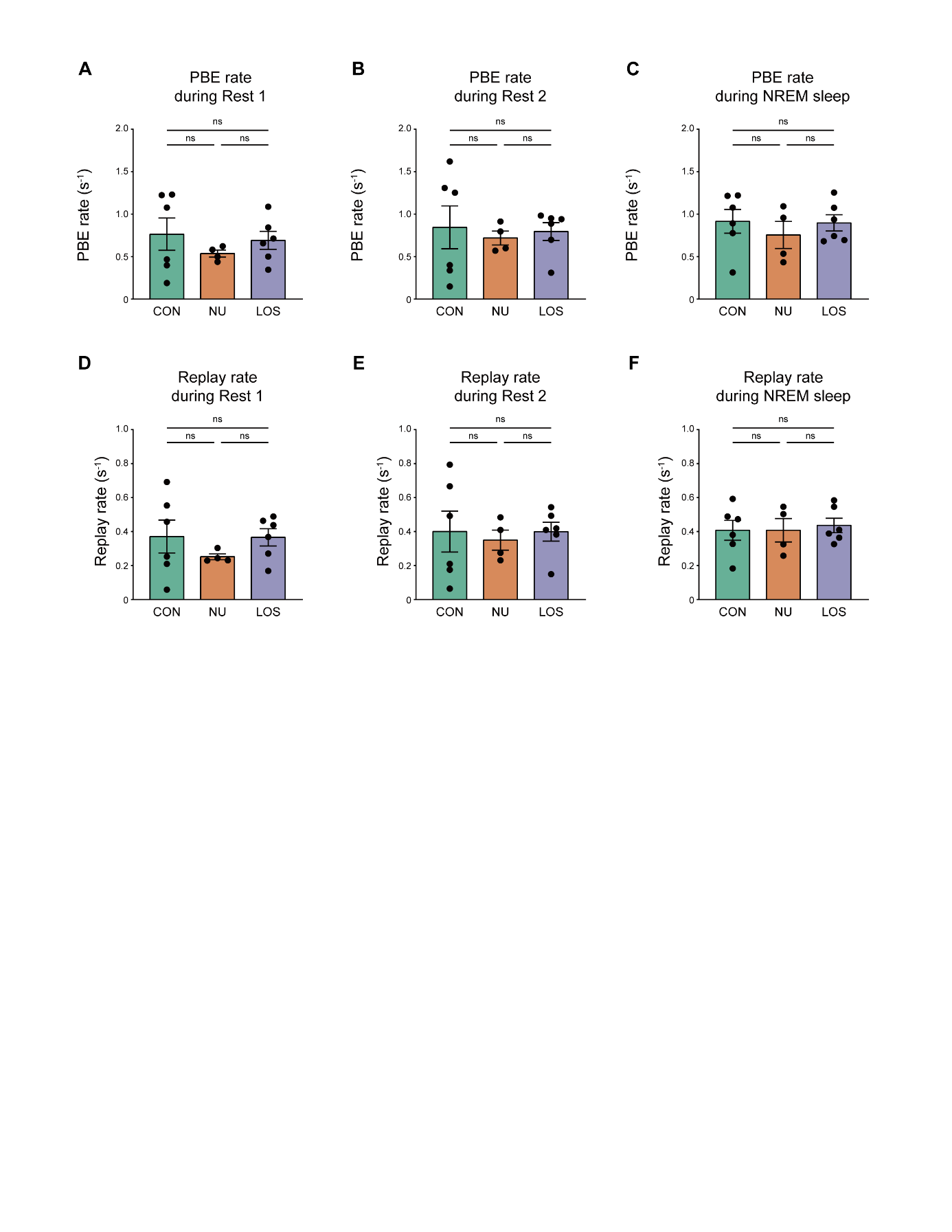


Figure S9. PBE rates and replay rates during offline sessions stayed unchanged in the NU period. (A-C) PBE rates during Rest 1 session (A), Rest 2 session (B) and NREM sleep (C) in CON, NU and LOS periods. One-way ANOVA with Tukey post hoc analysis, Rest 1 session: F (2, 13) = 0.5740, p = 0.5769, CON vs. LOS: p = 0.5506, CON vs. REV: p = 0.9241, LOS vs. REV: p = 0.7526; Rest 2 session: F (2, 13) = 0.1041, p = 0.9018; CON vs. LOS: p = 0.8925, CON vs. REV: p = 0.9782, LOS vs. REV: p = 0.9586; Sleep session: F (2, 13) = 0.3897, p = 0.6849, CON vs. LOS: p = 0.6909, CON vs. REV: p = 0.9938, LOS vs. REV: p = 0.7469. Data are presented as mean ± SEM (n = 6 rats). (D-F) Replay rates during Rest 1 session (D), Rest 2 session (E) and NREM sleep (F) in CON, NU and LOS periods. One-way ANOVA with Tukey post hoc analysis, Rest 1 session: F (2, 13) = 0.7348, p = 0.4985, CON vs. LOS: p = 0.5286, CON vs. REV: p = 0.9989, LOS vs. REV: p = 0.5521; Rest 2 session: F (2, 13) = 0.08617, p = 0.9180, CON vs. LOS: p = 0.9258, CON vs. REV: p > 0.9999, LOS vs. REV: p = 0.9287; Sleep session: F (2, 13) = 0.09427, p = 0.9106, CON vs. LOS: p > 0.9999, CON vs. REV: p = 0.9193, LOS vs. REV: p = 0.9377. Data are presented as mean ± SEM (n_CON_ = 6 rats, n_NU_ = 4 rats, n_LOS_ = 6 rats). ***, p < 0.001; **, p < 0.01; *, p < 0.05; ns, p > 0.05.


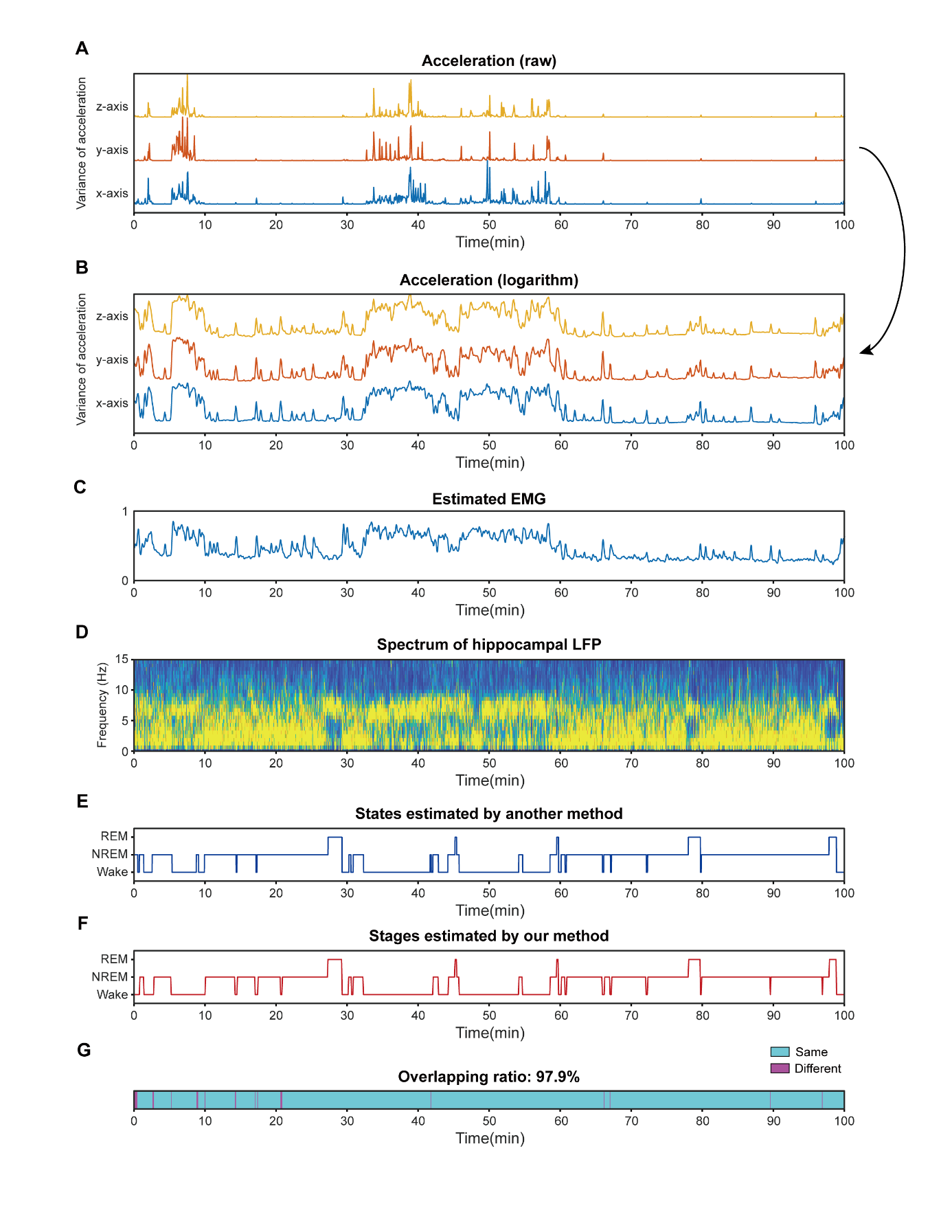


Figure S10. Verification of the sleep analysis method. (A) Variances of three-axis accelerometer data. (B) Logarithm of the variance of the accelerometer data. (C) Estimated EMG derived from LFP data recorded across different layers of the dCA1 brain region. (D) Power spectrum of LFP data recorded in the dCA1 brain region. (E, F) Awake and sleep states classification based on another widely used sleep analysis method (SleepScoreMaster in Buzcode, E) and our method (F). (G) Similarity between the results of the two analysis methods.
